# Anthropic cut marks in extinct megafauna bones from the Pampean region (Argentina) at the last glacial maximum

**DOI:** 10.1371/journal.pone.0304956

**Published:** 2024-07-17

**Authors:** Mariano Del Papa, Martin De Los Reyes, Daniel G. Poiré, Nicolás Rascovan, Guillermo Jofré, Miguel Delgado

**Affiliations:** 1 Facultad de Ciencias Naturales y Museo, División Antropología, Universidad Nacional de La Plata, La Plata, Argentina; 2 Facultad de Ciencias Naturales y Museo, División Paleontología Vertebrados, Universidad Nacional de La Plata, La Plata, Argentina; 3 Instituto Antártico Argentino (IAA), 25 de mayo 1143, San Martín, Provincia de Buenos Aires, Argentina; 4 Consejo Nacional de Investigaciones Científicas y Técnicas (CONICET), Provincia de Buenos Aires, Argentina; 5 Centro de Investigaciones Geológicas (CIG), CONICET—UNLP, Diagonal 113 n°275, La Plata, Argentina; 6 Institut Pasteur, Université de Paris Cité, CNRS UMR 2000, Microbial Paleogenomics Unit, F-75015, Paris, France; 7 Repositorio Paleontológico Ramón Segura, Merlo, Provincia de Buenos Aires, Argentina; 8 Ministry of Education Key Laboratory of Contemporary Anthropology and Collaborative Innovation Center of Genetics and Development, School of Life Sciences and Human Phenome Institute, Fudan University, Shanghai, China; University of Michigan, UNITED STATES

## Abstract

The initial peopling of South America is a topic of intense archaeological debate. Among the most contentious issues remain the nature of the human-megafauna interaction and the possible role of humans, along with climatic change, in the extinction of several megamammal genera at the end of the Pleistocene. In this study, we present the analysis of fossil remains with cutmarks belonging to a specimen of *Neosclerocalyptus* (Xenarthra, Glyptodontidae), found on the banks of the Reconquista River, northeast of the Pampean region (Argentina), whose AMS ^14^C dating corresponds to the Last Glacial Maximum (21,090–20,811 cal YBP). Paleoenvironmental reconstructions, stratigraphic descriptions, absolute chronological dating of bone materials, and deposits suggest a relatively rapid burial event of the bone assemblage in a semi-dry climate during a wet season. Quantitative and qualitative analyses of the cut marks, reconstruction of butchering sequences, and assessments of the possible agents involved in the observed bone surface modifications indicate anthropic activities. Our results provide new elements for discussing the earliest peopling of southern South America and specifically for the interaction between humans and local megafauna in the Pampean region during the Last Glacial Maximum.

## Introduction

The timing, pattern, and process of the early peopling of South America at the end of the Pleistocene are highly debated topics since the mid-19^th^ century. During these early stages, research efforts were focused in the co-occurrence of human remains and extinct mammals along with the eventual presence of hearths and lithic artifacts. Indeed, the earliest research dating back to 1880 proposed the coexistence of humans and extinct megafauna (referred as Pliocene fauna) on the basis of cultural evidence, alleged anthropic modifications of fossil bones and the geological context of the deposits [1]. This research was later revised or even discredited in the early 20^th^ Century [2], and the investigation of the early peopling processes progressively lost the interest of scholars working in southern South America and strongly influenced the development of the regional archaeological research throughout the 20^th^ Century, including the Pampean region [3].

The advent of new ^14^C dating technologies, theoretical frameworks, and methodological approaches (including ancient DNA techniques) revived the interest of the initial peopling of the Americas, which extended also to the case of the Southern Cone [4–6]. Despite major advances regarding our understanding of when and how humans entered the subcontinent, their biological and cultural diversity, how they adapted to diverse landscapes, environmental scenarios, and their routes of dispersal [7–10], the evidence of the first human populations entering the subcontinent and their interactions with local megafauna continue to be scarce, with the archaeological record fragmented and asymmetrically distributed in spatial terms [11–13].

**Fig 1:**
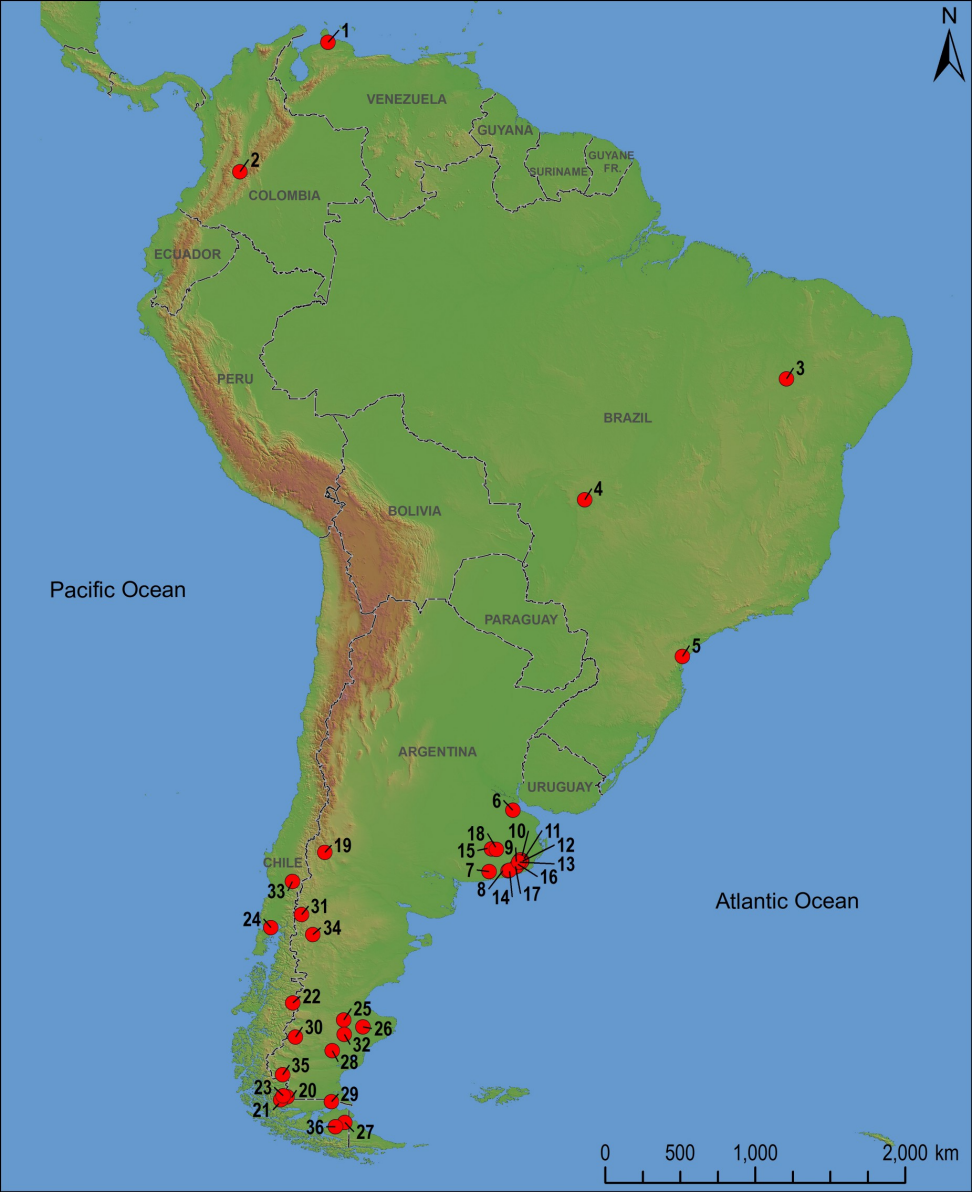
Map indicating the geographical location of archaeological sites with evidence of humans and extinct fauna across South America mentioned in the text corresponding to late Pleistocene and early Holocene occupations (dates calibrated at 2σ). 1) Taima Taima (14,200 ± 300–12,980 ± 85 ^14^C YBP [18,105–16,405–15,742–15,226 cal YBP]); 2) Tibitó (11,740 ± 420 ^14^C YBP [14,909–12,784 cal YBP]); 3) Toca da Janela da Barra do Antonião (9670 ± 140 ^14^C YBP [11,225–10,564 cal YBP]); 4) Santa Elina (23,120 ± 260–10,120 ± 60 ^14^C YBP [27814–26,617–11,870–11,137 cal YBP]); 5) Abismo Ponta da Flecha (11,380 ± 40–11,090 ± 40 ^14^C YBP [13,310–13,168 cal YBP]); 6) Río Reconquista I (17,397 ± 52 ^14^C YBP [21,090–20,811 cal YBP]); 7) Arroyo Seco 2 (12,240 ± 110–11,190 ± 110 ^14^C YBP [14,822–13,799–13,252–12,793 cal YBP]); 8) Paso Otero 5 (10,440 ± 100–10,210 ± 50 ^14^C YBP [12,617–11,889–11,973–11,525 cal YBP]); 9) Cerro La China (10,804 ± 75–10,525 ± 74 ^14^C YBP [12,892–12,520–12,689–12,091 cal YBP]); 10) Tixi cave (10,375 ± 90–10,045 ± 95 ^14^C YBP [12,587–11,845–11,836–11,241 cal YBP]); 11) Los Pinos 10,465 ± 65–8750 ± 160 ^14^C YBP [12,610–12,018–10,197–9469 cal YBP]); 12) Amalia site 2 (10,425 ± 75 ^14^C YBP [12,597–11,955 cal YBP]); 13) Burucuyá cave (10,000 ± 120 ^14^C YBP [11,869–11,190 cal YBP]); 14) Paso Otero 4 (9283 ± 83–7314 ± 73 ^14^C YBP [10,651–10,237–8309–7941 cal YBP]); 15) Campo Laborde (8090 ± 190–7750 ± 250 ^14^C YBP [9438–8465–9254–8013 cal YBP]); 16) Lobería 1 Sitio 1 (9878 ± 81 ^14^C YBP [11,628–10,896 cal YBP]); 17) Los Helechos (9640 ± 40 ^14^C YBP [11,166–10,763 cal YBP]); 18) La Moderna 8356 ± 65–7448 ± 109 ^14^C YBP [9482–9036–8407–8013 cal YBP]); 19) Huenul cave (13,840 ± 56–11,841 ± 56 ^14^C YBP [16,984–16,535–13,788–13,512 cal YBP]); 20) Milodón cave (13,630 ± 50–12,000 ± 50 ^14^C YBP [16,605–16,241–14,034–13,614 cal YBP)]; 21) Lago Sofía 4 cave (13,400 ± 90–11,590 ± 100 ^14^C YBP [16,343–15,789–13,600–13,190 cal YBP]); 22) Las Guanacas rockshelter (13,275 ± 50 ^14^C YBP [16,077–15,713 cal YBP]); 23) Lago Sofía 1 cave (12,290 ± 490 ^14^C YBP [15,788–13,195 cal YBP]); 24) Monte Verde (12,980 ± 40–11,959 ± 33 ^14^C YBP [15,639–15,295–14,010–13,608 cal YBP]); 25) Los Toldos cave 3 layer 11 (12,600 ± 650 ^14^C YBP [16,656–13,243 cal YBP]); 26) Piedra Museo-AEP-1 layer 6 (12,890 ± 60–11,000 ± 50 ^14^C YBP [15,580–15,162–13,065–12,764 cal YBP]); 27) Tres Arroyos rockshelter (11,880 ± 250–11,280 ± 110 ^14^C YBP [14,793–13,175–13,352–12,916 cal YBP]); 28) Casa del Minero 1 cave (11,000 ± 55–10,970 ± 55 ^14^C YBP [13,067–12,763–13,000–12,746 cal YBP]); 29 Fell cave (11,000 ± 160–10,720 ± 300 ^14^C YBP [13,178–12,637–13,230–11,521 cal YBP]); 30) Cerro Casa de Piedra 7 (10,530 ± 620 ^14^C YBP [13,505–10,511 cal YBP]); 31) El Trébol rockshelter (10,600 ± 100–10,570 ± 130 ^14^C YBP [12,741–12,091–12,736–12,000 cal YBP]); 32) La María Túnel cave (10,400 ± 100 ^14^C YBP [12,608–11,847 cal YBP]); 33) Marifilo Rockshelter 1 (10,410 ± 70–8420 ± 40 ^14^C YBP [12,582–11,942–9524–9284 cal YBP]); 34) Epullán Grande cave (9970 ± 100–7550 ± 70 ^14^C YBP [11,760–11,193–8445–8175 cal YBP]); 35) Chorrillo Malo 2 (9740 ± 50–9690 ± 80 ^14^C YBP [11,235–10,798–11,211–10,744 cal YBP]); 36) Marazzi rockshelter (9590 ± 210 ^14^C YBP [11,246–10,239 cal YBP]).

The association of humans and extinct animals, large-bodied mainly, has been documented at some early sites across South America including (Fig 1): Tibitó (Sabana de Bogotá, Colombia) [14]; Taima Taima (Venezuela) [15]; Toca da Janela da Barra do Antonião, Abismo Ponta da Flecha, Santa Elina rockshelter (Brazil) [16–19]; Arroyo Seco 2, Campo Laborde, La Moderna, Cerro La China, Tixi cave, Los Pinos, Amalia site 2, Cueva Burucuya, Lobería 1 site 1, Los Helechos, Paso Otero 4 and Paso Otero 5 (Pampean Region, Argentina) [20–31]; Huenul cave, Los Toldos cave 3, Piedra Museo-AEP-1, Casa del Minero 1 cave, Cerro Casa de Piedra 7, El Trébol rockshelter, Túnel cave, Epullán Grande cave, Chorrillo Malo 2 (Argentine continental Patagonia) [32–40]; Las Guanacas rockshelter, Monte Verde, Fell cave, Marifilo rockshelter, Milodón cave, Lago Sofía-1 cave, Lago Sofía 4 cave (Chilean continental Patagonia) [41–47]; Marazzi rockshelter and Tres Arroyos rockshelter (Chilean insular Patagonia) [48,49]. In this sense, the Pampean region has played a central role in the discussions about the timing and pattern of the peopling of the Southern Cone during the late Pleistocene, especially regarding the interaction between humans and megafauna, the spatiotemporal distribution of different megamammal genera and their exploitation by humans in the context of broad-spectrum subsistence strategies [11,20–31]. In general, in the Argentine Pampas the archaeological record of the extinct megafauna is regionally restricted [50] and some areas (e.g., the North) still lack evidence of it.

The association between humans and megafauna (*sensu* [11]:145) can be supported in the form of (1) physical associations, which simply refer to bones and tools found side by side, or in the same deposit; and (2) behavioral associations, which require the demonstration of human activities related to megafauna. A trademark of human behavioral contexts is the presence of cut marks in bones, which reflect direct interactions, and represents the most expected evidence for megafaunal bones processed by humans. The challenge for such evidence is to demonstrate that it was human made, discarding postmortem and perimortem modifications from non-human agents.

**Fig 2:**
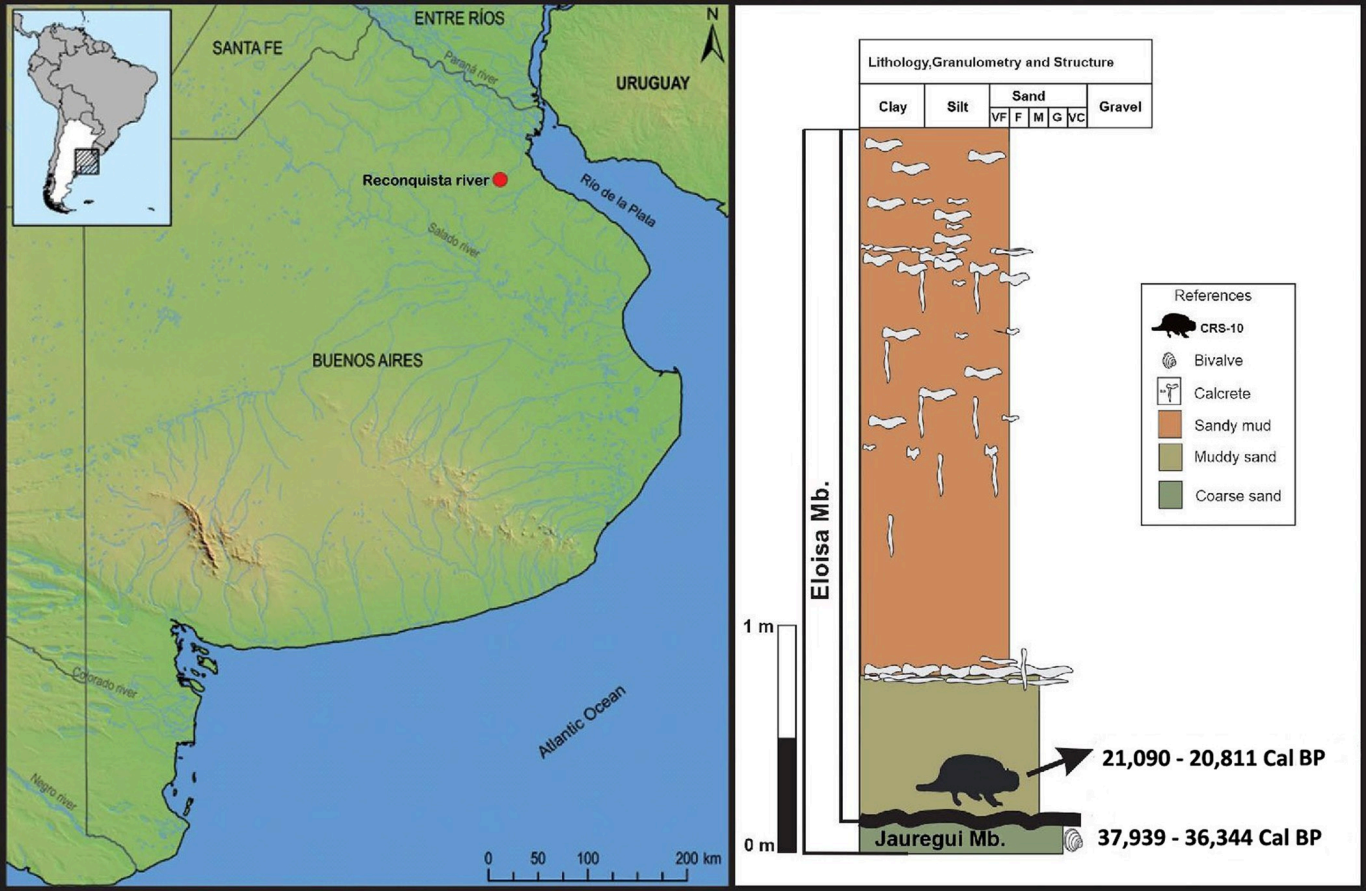
Map showing the location of the site investigated. A litho-stratigraphic profile of the site where the CRS-10 specimen was found (banks of the Reconquista River), including the calibrated radiocarbon dates obtained. Base map: MDE-Ar v2.1 hillshade and 1:250000 vector layers from the IGN (Instituto Geográfico Nacional, República Argentina).

During surveys performed at the southern margin of the Reconquista River, the northern area of the Pampean region (Fig 2), fossil bones of extinct megafauna were uncovered. Subsequent analyses revealed cut marks but no physical associations (*sensu* [11]), that could be potentially linked to human activities, were identified in the site. The bone surface modifications (BSMs) observed in several anatomical units required a detailed analysis taking into account the context of the finding and the taphonomic history of the deposits, that is, the potential agents involved in the site formation processes, especially those agents acting during perimortem and postmortem moments, to eventually defend a behavioral association (*sensu* [11]).

Here, we present the results of multiple analyses performed in the fossil remains of a *Neosclerocalyptus* sp. (Xenarthra, Glyptodontidae) specimen (CRS-10), which altogether represents what we consider one of the earliest evidence of the interaction between humans and local megafauna in South America at the end of the Pleistocene (see also [19]).

## Materials and methods

**Fig 3:**
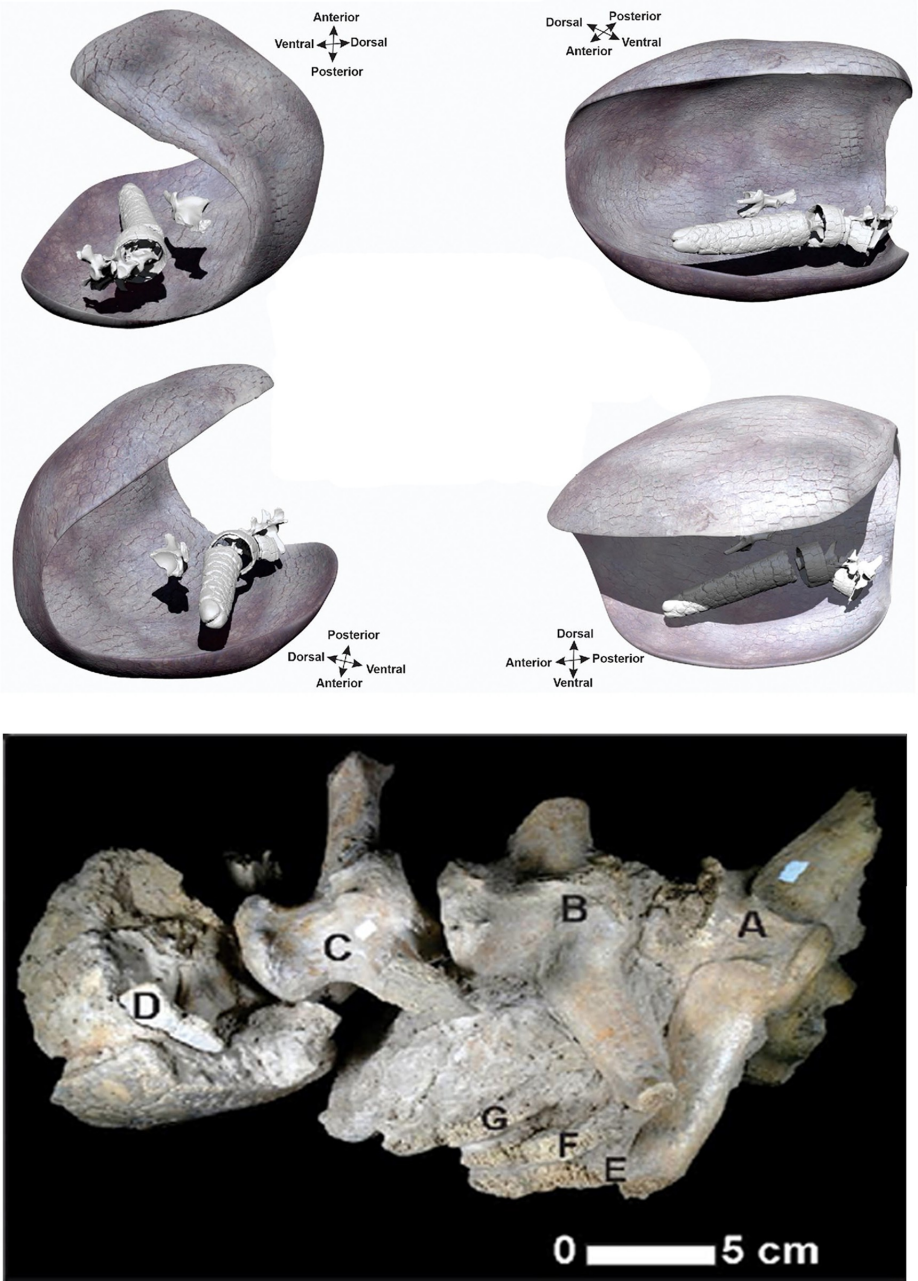
A) Reconstruction of the 3D scanned skeletal elements found and the carapace of the CRS-10 specimen in anatomical position. B) detail of the *in situ* articulated vertebrae. A: caudal vertebra 4; B: caudal vertebra 5; C: caudal vertebra 6; D: caudal vertebra 7; E: fractured ring from caudal vertebra 4; F: fractured ring from caudal vertebra 5 and G: fractured ring from caudal vertebra 6.

The remains of the specimen CRS-10 are under the guard of the Municipal Paleontological Repository of Merlo "Colección Ramón Segura" (Buenos Aires province, Argentina). They are represented by several skeletal elements of the posterior portion of the specimen, among which are fragments of the right side portion of the carapace, the synsacrum fragmented including the acetabular cavity and a part of the ilium, right ischium and pubis, caudal rings, four caudal vertebrae (4,5,6, and 7) and the caudal tube (Fig 3). As shown below, we scanned these materials using a high-resolution Artec Space Spider 3D scanner and the 3D scans are at the División Antropología (FCNyM, UNLP) available upon reasonable request.

We obtained from the Province Government through the Centro de Registro del Patrimonio Arqueológico y Paleontológico (CREPAP) the license 2020-3-A-207-2 to perform the archaeological and paleontological recovery of the materials here investigated. Likewise, we obtained the license F-2021-49010608-APN-MACN#CONICET from Museo Argentino de Ciencias Naturales-CONICET (National Government) to export some fragments of bone to perform radiocarbon dating. All necessary permits were obtained for the described study, which complied with all relevant regulations.

### Sediment analysis

**Fig 4:**
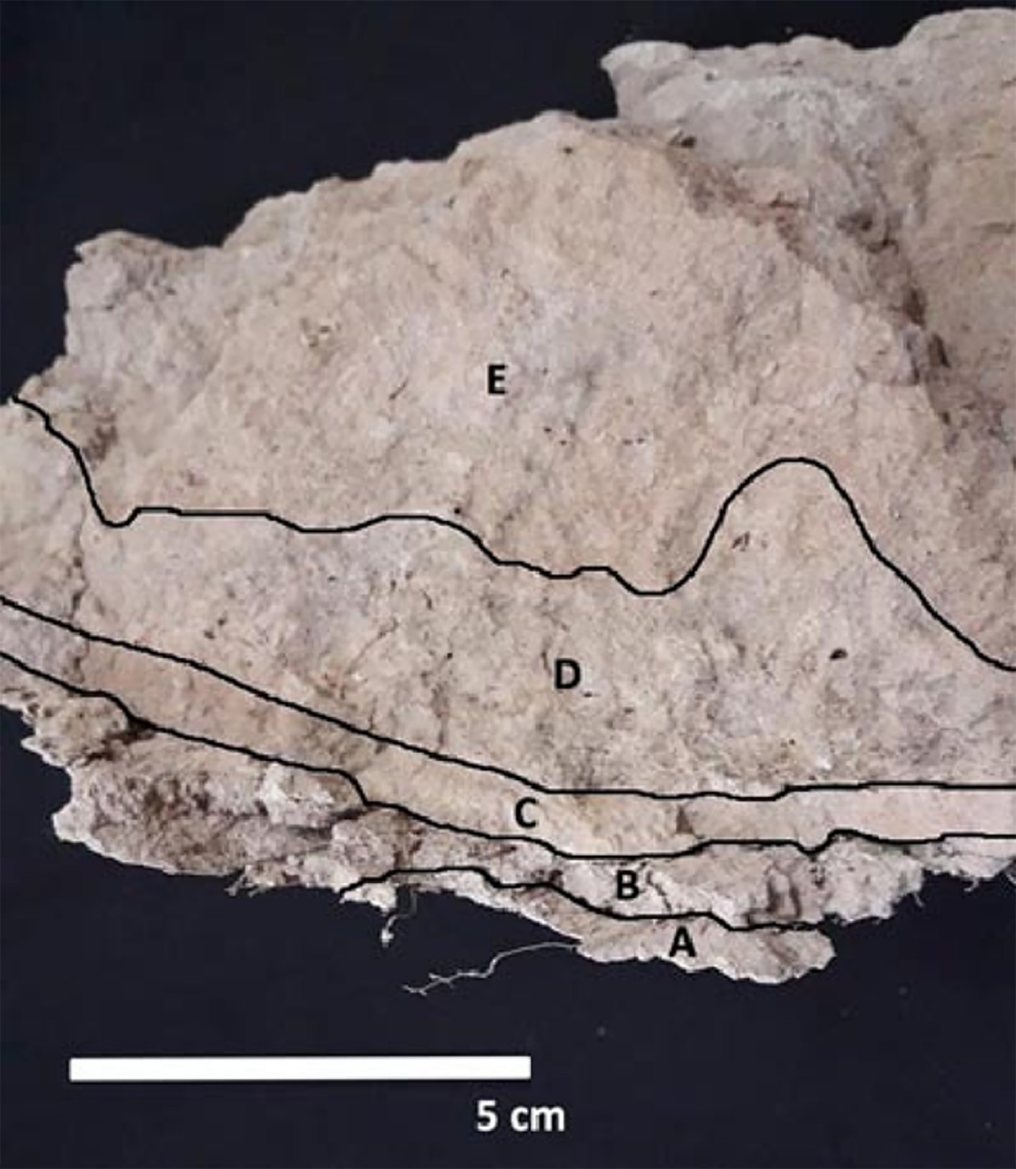
Sediment arrangement; A: First laminar structure; B: Second laminar structure; C: Third laminar structure; D: First massive structure; E: Second massive structure.

The analysis of sediments containing the caudal vertebrae, as well as in a posterior carapace fragment was carried out, with particular emphasis on the structure and granulometry of these sediments (Fig 4). Once the sediments containing the vertebrae and carapace were cleaned, they were examined in a 1 mm sieve to search for a range of evidence including lithic microdebris and small bone fragments. The anatomical units were analyzed by eye using a binocular magnifier (Nikon SMZ 800). In addition, we performed a more detailed examination through Scanning Electron Microscopy (SEM) in the Laboratory of Research in Metallurgical Physics “Ing. Gregorio Cusminsky” (LIMF, UNLP), using an FEI ESEM Quanta 200 with electron source from a tungsten filament, with accelerating voltage of 200 V to 30 kV. Through these procedures, the presence of different taphonomic agents that could have acted during perimortem and postmortem moments was sought. Chemical analyses were also performed using energy dispersive X-ray spectrometry (EDX). This technique is used for elemental analysis or chemical characterization of a range of samples, in this case fossil bones. It relies on an interaction between some source of X-ray excitation and a sample. EDX can be used to determine which chemical elements are present in a sample (qualitative analysis), and can be used to estimate their relative abundance (quantitative analysis). In quantitative analysis, the concentration of a specific element present in a sample is measured by the intensities of peaks. Since each element has a unique atomic structure allowing a unique set of peaks on its electromagnetic emission spectrum, in qualitative analysis, different X-ray peaks with specified positions in a spectrum are identified [51]. For this analysis we chose a fragment of the transverse apophysis of the fifth vertebrae (CV5) to investigate the chemical composition of dark colorations present in almost all bones found. This gives us details about the environmental conditions present during the formation of such a patina.

### Radiocarbon dating

To contextualize chronologically the fossil assemblage recovered, two radiocarbon dates were obtained. The first came from a fragment of the pelvis of the CRS-10 specimen (AMS) obtained at CIRAM SAS Lab (code CIRAM-2620) (Pessac–France). Given the absence of collagen, the date was determined from hydroxyapatite, the inorganic bone fraction a procedure widely validated in the ^14^C dating methods currently available [52,53].

### Sample preparation

Bone samples were chemically pretreated using the methodology applied by Cherkinsky et al. [54]. After washing the samples with water and cleaning the external surface of bone fragments, 4–5 g of the fragments were treated with 2 × 50 mL of 0.25 M sodium chlorite (24 hr, 20°C) in a centrifuge tube to remove organic residues. After that, the samples were washed in ultrapure water followed by treating the samples with 2 × 50 mL 1 M acetic acid (24 hr, 20°C) to remove exogenous carbonates and less well-crystallized crystallites of apatite. Finally, all samples were washed repetitively in ultrapure water again until a transparent liquid was observed and, after decantation, samples were oven-dried at 60°C overnight.

### Graphitization

The graphitization procedure of the samples was performed using Carbonate Handling System and Automated Graphitization Equipment (CHS-AGE-3, Ionplus AG). 600 mg of the crushed sample was weighed in a borosilicate vial, flushed with helium carrier gas, treated with 85% orthophosphoric acid, and kept at 70°C for 5 hr in the Carbonate Handling System. The CO2 released from the sample was carried by He gas to AGE-3 graphitization system through the online sulphur trap consisting of a heater with controlled temperature and a quartz tube filled with silvered cobaltous oxide granules. In the AGE-3 system, CO2 was trapped on the zeolite trap and thermally released into the reactor with an iron catalyst. The graphitization reaction of CO2 with H2 took place at 580°C for 2 hours. The AGE-3 system consists of 7 reactors in which graphitization takes place simultaneously.

### Radiocarbon analysis

Radiocarbon measurements were made with a 240 kV Single-Stage Accelerator Mass Spectrometer (SSAMS, NEC, USA) with 39 position Source of Negative Ions by Cesium Sputtering (SNICS). The background of measurements was estimated to be 2.45 × 10−3 fM (fraction of modern carbon) using phthalic anhydride. The IAEA-C2 and SIRI-K (carbonate) standards were used as reference materials. The ^14^C/^12^C ratio was measured with an accuracy better than 0.3%. For the isotopic fractionation correction, the ^13^C/^12^C ratio was used. Radiocarbon dates are reported as pMC (percent of modern carbon) and in years before 1950 (radiocarbon age BP). The correction factor used was a pMC value of 11.47 ± 0.07.

A second date was made on bivalve mollusks *Diplodon* sp. coming from the Jáuregui member, the level below the CRS-10 finding. The calibration was done using an estimated correction factor *δ*^*13*^*C* = pMC -8 ± 2‰ (error multiplying factor K = 1) performed at the Laboratorio de Radiocarbono del Centro de Investigaciones Geológicas, CONICET-Universidad Nacional de La Plata, La Plata, Argentina. (LATYR–UNLP). The treatment protocol for the mollusk shells was washing with water and then subjected to ultrasonic bath to remove traces of adhered material, then the specimens were treated with HCl solution to remove the 20% in wt of the surface carbonate, and the dried sample was transformed into benzene. The activity was measured by liquid scintillation spectrometry (LSC) with Packard Tricarb 3170TR/SL equipment. Ages are in radiocarbon years before present (1950 AD)—denominated conventional radiocarbon age- corrected by isotopic fractionation with *δ*^*13*^*C* values were estimated by table [55]. Both dates were calibrated using the rcarbon package [56] and the calibration curve SHcal 20 [57] at 2-sigma, 95.4% probabilities.

### Cut marks characterization

Cut marks found on bone surfaces of several anatomical units were characterized through distinct morphological attributes (Table 1) which allowed us to support or discard their anthropic nature on the basis of the criteria described below. In addition, Table 1 includes which cut marks were included or excluded in the linear (LM) and/or 2D geometric morphometric analyses (GMM).

**Table 1:** Linear mark characterization through distinct morphological attributes.

Skeletal element		SCS	ACS	ChM	HC	M	SE	Db	Sl	LM	GMM
Pelvis	1	X			X					X	
	2		X				X				
CV4	3		X					X		X	X
	4			X						X	X
	5	X									
	6			X							
	7	X								X	
	8	X								X	
CV5	9				X	X			X	X	X
	10		X							X	
	11		X								
	12	X									
CV6	13	X			X					X	X
	14	X								X	X
	15	X								X	X
	16	X								X	X
	17	X								X	X
	18	X								X	X
	19	X									X
	20	X									
	21	X									
	22	X									
	23		X			X					
	24		X			X					
CR1	25	X								X	X
	26	X								X	X
	27	X								X	X
	28	X								X	
	29	X									
	30	X									
CR2	31									X	X
	32									X	

SCS: symmetrical cross-section; ASC: asymmetrical cross-section; ChM: Chop mark; HC: Hertzian cones; M: microstriations; SE: shoulder effect; Db: displaced bone; Sl: supine line; LM: linear morphometrics; GMM: geometric morphometrics.

### Butchering sequences and patterns

Refers to the non-random distribution of cut marks on faunal bone surfaces explained by human agency. Although numerous variables are involved in the processes of bone surface modifications formation, some authors have argued that it is possible to infer past patterns of behavior (i.e., derived from butchering processes) through the investigation of the anatomical distribution of cut marks as well as their frequency, and size/shape variables [11,58–61].

#### Form

Cutting marks made with lithic instruments usually exhibit a V-shaped cross-section (wide or narrow), while teeth of carnivores develop a U-shaped cross-section [62–65]. Cutting marks can be expressed with both sides having a symmetrical cross-section [62–65] but in some cases, an asymmetric cross-section is present with a steeper slope than another depending on the angle of the tool regarding the bone surface [66,67].

#### Microstriations

Can be attributed to the movement of rocks on the bone produced by their displacement (e.g., mechanical action of trampling, transport, etc) or by retouched edges of lithic instruments [68].

#### Shoulder effect

Pseudo cut marks parallel to the primary striae [69].

#### Displaced bone

Displaced bone may build up on a raised shoulder of bone that runs along the linear mark which, in the absence of abrasion, may remain *in situ* [68].

#### Hertzian cones

elevations to the main striae of triangular morphology, produced by the differential pressure exerted on the bone surface when cutting and by the resistance of the bone surface to cutting [70].

#### Supine line

The directionality of the cut may also be indicated by the supination of the end of the linear mark where the striae is curved to one side by bone contact when the cutting object ceases to mark [68].

#### Chop marks

Mark with usually deep penetration into the bone with a smooth sloping side showing the direction of the cut made by a sharp stone tool edge striking the bone surface [68]

#### Stratigraphy

The stratigraphic scheme used in the present study was proposed by Toledo [71] who, on the basis of several detailed geological surveys and ^14^C, OSL, and ESR dating, established the most recent and complete stratigraphic scheme available for the study area. In Fig 2. and in the results section, we present in detail the geological context of the deposits.

### Quantitative analysis of cut marks

We performed an analysis of bone surface modifications (BSMs) of the CRS-10 specimen to evaluate quantitatively the cut marks presented on the specimen. Accordingly, we used linear morphometric (LM) and 2D geometric morphometric analyses (GMM). In the present study, we followed the recommendations of Courtenay and colleagues [60]. This is a new approach recently developed to deal with the correct assessment and classification of BSMs in the context of anthropic activities [60,61,72–74]. Although many methodologies (including 3D approaches) exist to quantify the size and shape of cutmarks [60,61,72–74], we focused on 2DGMM because we investigated cutmark cross-sections whose shape changes are best represented in a 2D morphospace and because the sample size of cutmarks is relatively small implying that the use of 3D data will produce much more variables than cases adding noise to the statistical analyses. Likewise, previous studies [60] showed clearly that the use of sophisticated 3D methods does not contribute to an improvement in accuracy.

We included 19 out of 32 linear marks that presented expected characteristics (e.g., depth, size, direction, angle, etc.) to be included in the analyses. We discarded 13 cut marks for which we couldn’t obtain reliable data on thickness, depth, and angle because the measurement was short and shallow and the width of the incision was very large and asymmetric. Despite no strict relationship between the morphological attributes mentioned above and the quantitative analyses (i.e., they are independent), we focused mostly but not exclusively on V-shaped cutting marks cross-sections. Accordingly, this kind of analysis does not account for all morphological attributes mentioned in Table 1.

First, we digitized the cut marks with a high-resolution 3D Artec Space Spider scanner (Artec 3D, Santa Clara, California). The scans were conducted at 0.1 mm 3D resolution and 0.05 mm 3D accuracy. The Artec Studio software v15 was used for data processing using the manufacturer’s recommendations and specifications. High-resolution 3D models of the cut marks were then exported as.*OBJ* files including volume, color, and texture. Given the high resolution of the scanner, we recovered the detailed topography of the external bone surface with up to 1.8 million of points providing highly detailed 3D models.

Once the 3D models were obtained we used Avizo 7.1 (Visualization Sciences Group, USA) to define marks profiles along the groove. Following [74] cut mark sections were obtained at mid-length (always between 30% and 70% of the mark length) to perform the 2D analysis. We obtained according to Courtenay and colleagues [60:6] seven linear measurements in mm (WIS width of the incision at the surface; WIM width of the incision at the mean; WIB width of the incision at the bottom; D depth of the incision; OA opening angle of the incision; LDC left depth of the incision convergent and RDC right depth of the incision convergent) in ImageJ 1.5e [75] on the 2D cross-section of each mark (S1 Fig), which indicate the thickness, depth, and angles of the mark. For comparative purposes, we used two datasets of measurements obtained experimentally by Courtenay et al [60] at two different cutting angles 45° (cuts N = 60) and 90° (slices N = 60) to evaluate the similarities and differences with those obtained from the CRS-10 specimen through a principal component analysis or PCA (using the covariance matrix) under the assumption that close similarities, across the multivariate morphospace, between both sets of measurements help to support the anthropic origin of the marks found in the fossil assemblage here investigated. Following Courtenay et al [60], we also used a Multivariate Analysis of Variance (MANOVA) and a jackknife cross-validated Linear Discriminant Analysis (LDA) to assess the level of differentiation between cuts and slices derived from the experimental and fossil datasets. First, we conducted a LDA using three distinct datasets cuts (45°), slices (90°) and those obtained from the CRS-10 specimen to classify them into two categories: cutting and slicing cutmarks through the use of the highest and second-highest posterior probabilities. Subsequently, we conducted a MANOVA to evaluate the presence of two distinct cutting mark groups (cuts and slices) in the combined dataset through the comparison of their means. Finally, we applied a LDA to stablish differences between the two cutting mark groups through the computation of the confusion matrix and percentages of correct classification. The MANOVA and LDA results are reported including and excluding one of the measurements (OA the opening angle of the incision) given its major impact in the variance explained (see [60]). We used the Manova.rm, Mass and Factoextra R packages [76–78] to conduct the multivariate analyses mentioned.

Finally, we used geometric morphometric methods to evaluate in detail the shape variation of the CRS-10 cutmarks. We employed TPSDig2 (v.2.3.1) [79] to digitize seven landmarks (S1 Fig). The TPS files were imported into the R 4.1.0 software [80] to conduct a 2D geometric morphometric analysis using landmark coordinates through the package geomorph (4.1.2) [81]. A Generalized Procrustes Analysis (GPA) and a subsequent PCA were carried out using shape variables, and the plots were obtained using the package ggplot2 [82]. Procrustes coordinates and the shape changes were represented as TPS-grids diagrams. TPS-grids show deformations and color-coded Jacobian expansion/contraction factors, which measure the degree of local expansion or contraction of the grid. Jacobian factors computed in PAST 4.7.0 [83] help with the visualization of shape changes because they highlight the region of the TPS-grid whose deformation corresponds to expansions or contractions according to a system of color coding.

## Results

### Geological context & taxonomy of the CRS-10 specimen

Fossil remains of a *Neosclerocalyptus* sp. (Xenarthra, Glyptodontidae) specimen were found in sediments corresponding to the Eloisa Member (~ 30,000–16,000 YBP) [71] of the Luján Formation (~ 44,000–12,000 YBP) [66] of the Reconquista River, on the margin belonging to the town of Merlo (Buenos Aires, Argentina). The ravines that emerge in this sector of the river reach an approximate level of 4.5 meters thick. The profile base begins at the top of the Jáuregui Member (~ 39,000–29,000 YBP) [84], integrated by greyish muddy sands (2.5Y/8/1), with a prismatic texture and blackish complexions. On the discordance of the Jáuregui Member [84] there is a muddy sand layer that reaches a thickness of 0.8 meters, massive, of brown color (75YR/7/4), which in its lower part contains speckles of green and dark grey (oxides of Mn and Fe). This level contains the CRS-10 specimen which has isolated rhizoconcretions up to 4 centimeters in diameter with preferential horizontal development. At the top of this layer a discontinuous level (10 centimeters thick) comprised of carbonates is identified. Above this level is the upper section of the Eloisa Member, which is made up of massive sandy silt of brown color (7.5YR/ 7/2), presenting rhizoconcretions up to 1.5 centimeters in diameter and elongated vertically. There are also abundant carbonate nodules (tosca concentrations). The upper portion of the Eloisa Member and the entire El Rincon Member (~ 16,000–12,000 YBP) are not present (Fig 2).

**Fig 5:**
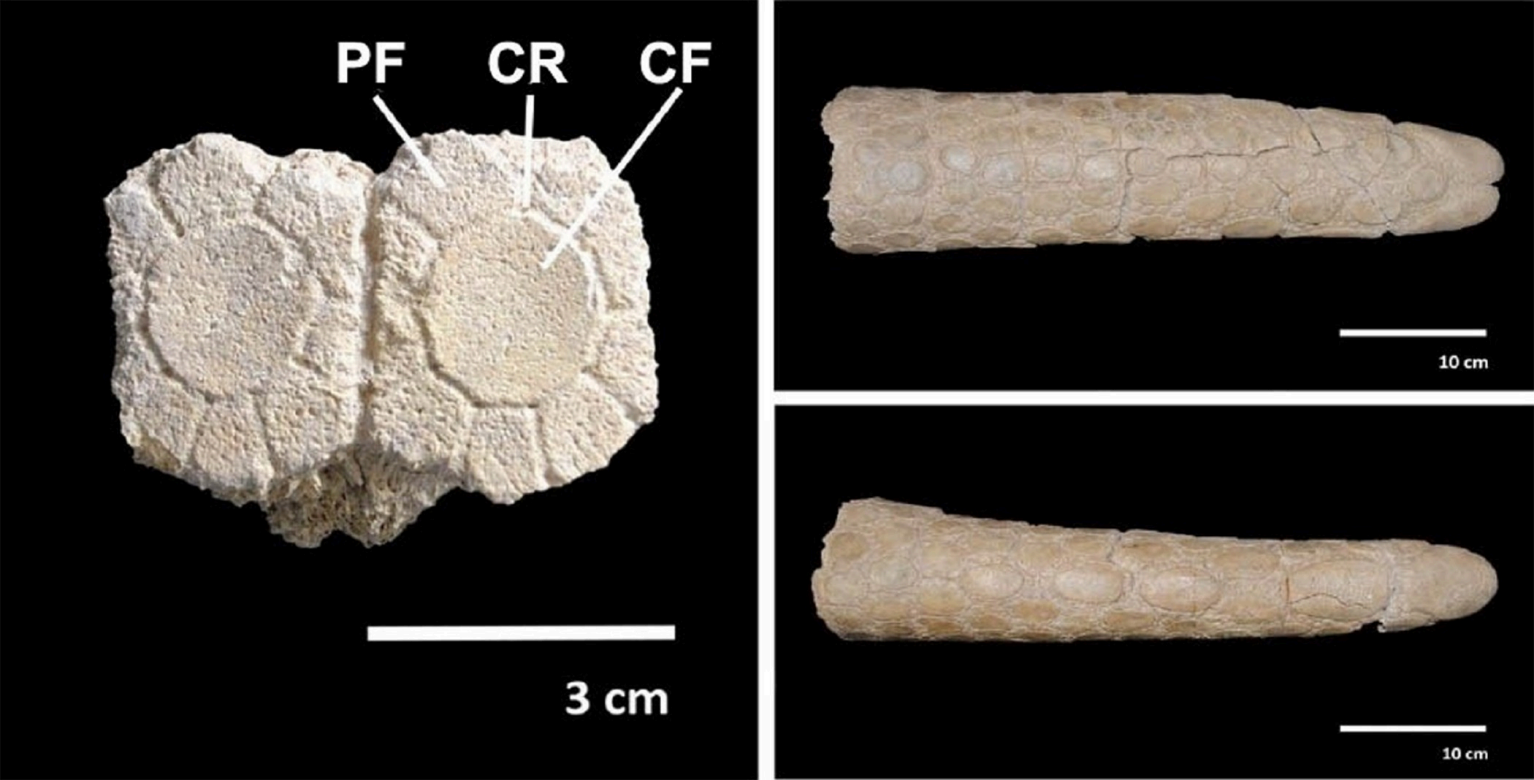
Left: osteoderms of the lateral dorsal portion of the carapace with the typical figure in “rosette”. CF Central figure, PF peripheral figurine, RG ring groove. Right: caudal tube in lateral and ventral views.

From a taxonomic point of view, the specimen CRS-10 is assigned to the genus *Neosclerocalyptus* based on a morphological assessment. The osteoderms that were preserved from the right lateral portion of the carapace are characterized by having a central figure surrounded by a row of minor figures, defining the typical ornamentation in "rosette" (Fig 5). It was also observed that the annular grooves and radials in the cross-section have a parabolic shape. The caudal tube has ornamentation similar to those described above; its cylindrical shape is somewhat flattened ventrally back and shrinking distally where it ends in two large figures that cover the entire end of the tube [85]. Due to the absence of the skull, which contains the specific diagnostic characters, classification at the species level was not possible.

### Radiocarbon chronology

The first radiocarbon date obtained from the pelvis of the CRS-10 specimen (code CIRAM 2620) was 17,397 ± 52 YBP whose calibration is 21,090–20,811 cal YBP (95.4%) (two sigma) (S1 Table, S2 Fig). The second ^14^C date obtained on bivalves coming from the Jáuregui member, the level below containing the CRS-10 specimen was 31,970 ± 640 YBP (code LP- 3771) and its calibration was established at 37,939–36,344 cal YBP (S1 Table, S2 Fig).

### Taphonomy (deposits context)

The specimen of *Neosclerocalyptus* sp. at the time of burial was lying on its right side. All preserved elements corresponding to the tail were inside the carapace, and the articular arrangement of the caudal vertebrae formed a curve (Fig 3). The sediments recovered from the interior part of the carapace correspond to fine sands and clays of light brown color (7.5YR/7/4) with some grayish-greenish tints. Laminated structures and massive levels were identified. The laminate (clayey) was in contact with the interior surface of the carapace and above these laminates the sediments become sandy and of massive structure (Fig 4).

In glyptodontids and in *Neosclerocalyptus* in particular, the portions of the tail where the 4, 5, 6, and 7 caudal vertebrae are located (CV 4, CV 5, CV 6, and CV 7), are covered in their entire circumference by caudal rings and culminate in a caudal tube. The rings are formed by a double row of osteoderms. In specimen CRS-10, the caudal vertebrae 4, 5, 6, and 7 at the time of finding were articulated in anatomical position, observing the absence of the ventral portions of the ring in the caudal vertebrae 4, 5, and 6. The dorsal portions of the caudal rings corresponding to CV 4, CV 5, and CV 6 are superimposed (Fig 3). It should be noted that some of the missing osteoderms of the ventral portion of the rings were mostly disarticulated and located on one side of the vertebrae. Another element found on one side of the tail was a fragment of the pelvis (left). In sum, the CRS-10 specimen was found *in situ* in anatomical position with minimal displacement from its place of burial near a body of water but not within it.

### Taphonomic attributes

The entire surface of the bones has a fairly uniform light brown color. In addition to this tonality, dark spots were observed in some areas, which were analyzed by Energy Dispersive X-Ray Analysis (EDX) to identify the elemental composition of materials. This analysis revealed the presence of the elements of Fe and Mn. The low values of these manganese and iron oxides are due to the extremely thin and disordered nature of the particles that form these coatings (S2 Table, S3 Fig) [71].

The fossil skeletal assemblage does not show significant signs of weathering (pericondral scaling or deep cracking). Shallow incipient longitudinal cracks could be observed on the inner side of the left ilium. In the caudal vertebrae 4, 5, and 6 (CV 4, CV 5, and CV 6), these cracks were also observed but even less marked than those observed in the ilium. They are located on the transverse processes and some of them were filled with Mn and Fe oxide. Discoloration by chemical action produced by plant roots is recorded on the anterior ventral side proximal to the transverse process of CV 4. According to these analyses, the effects of pericondral scaling or deep cracking on the CR-10 specimen were negligible, with no evidence of trampling or erosion caused by the bearing.

### Linear marks on the CRS-10 specimen

**Fig 6:**
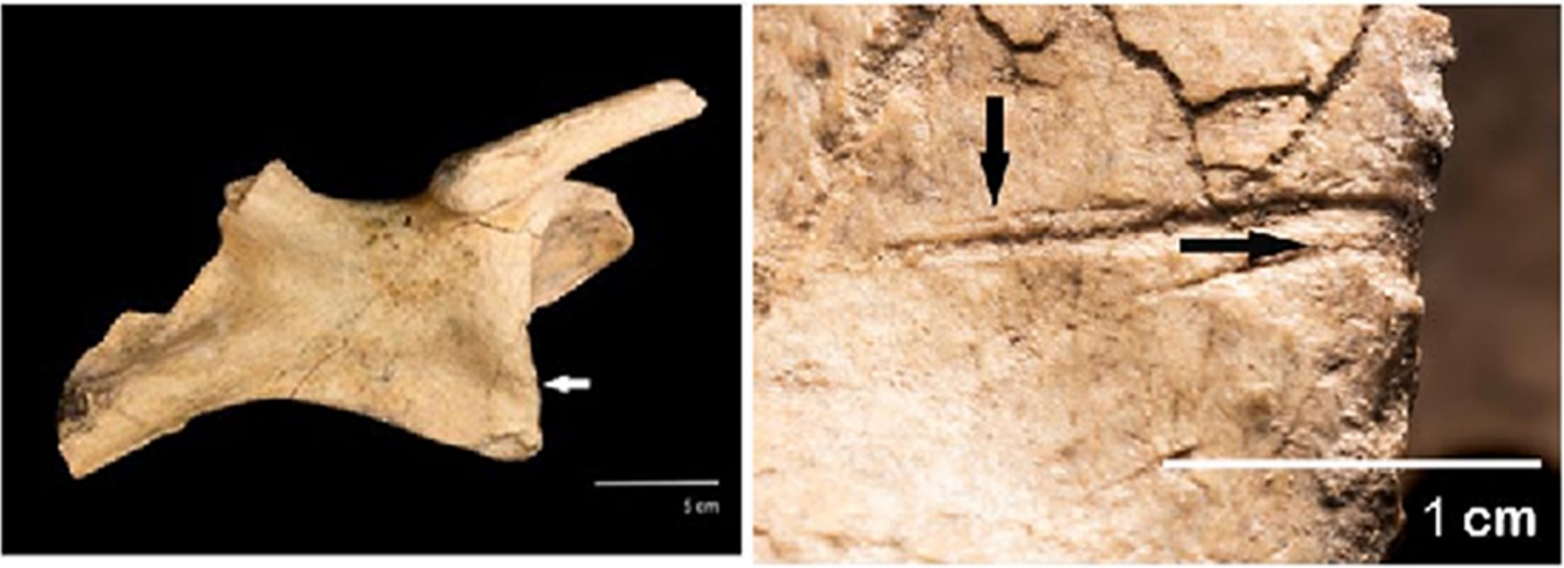
Marks with shoulder and hertzian cones in the pelvis of the CRS-10 specimen.

**Fig 7:**
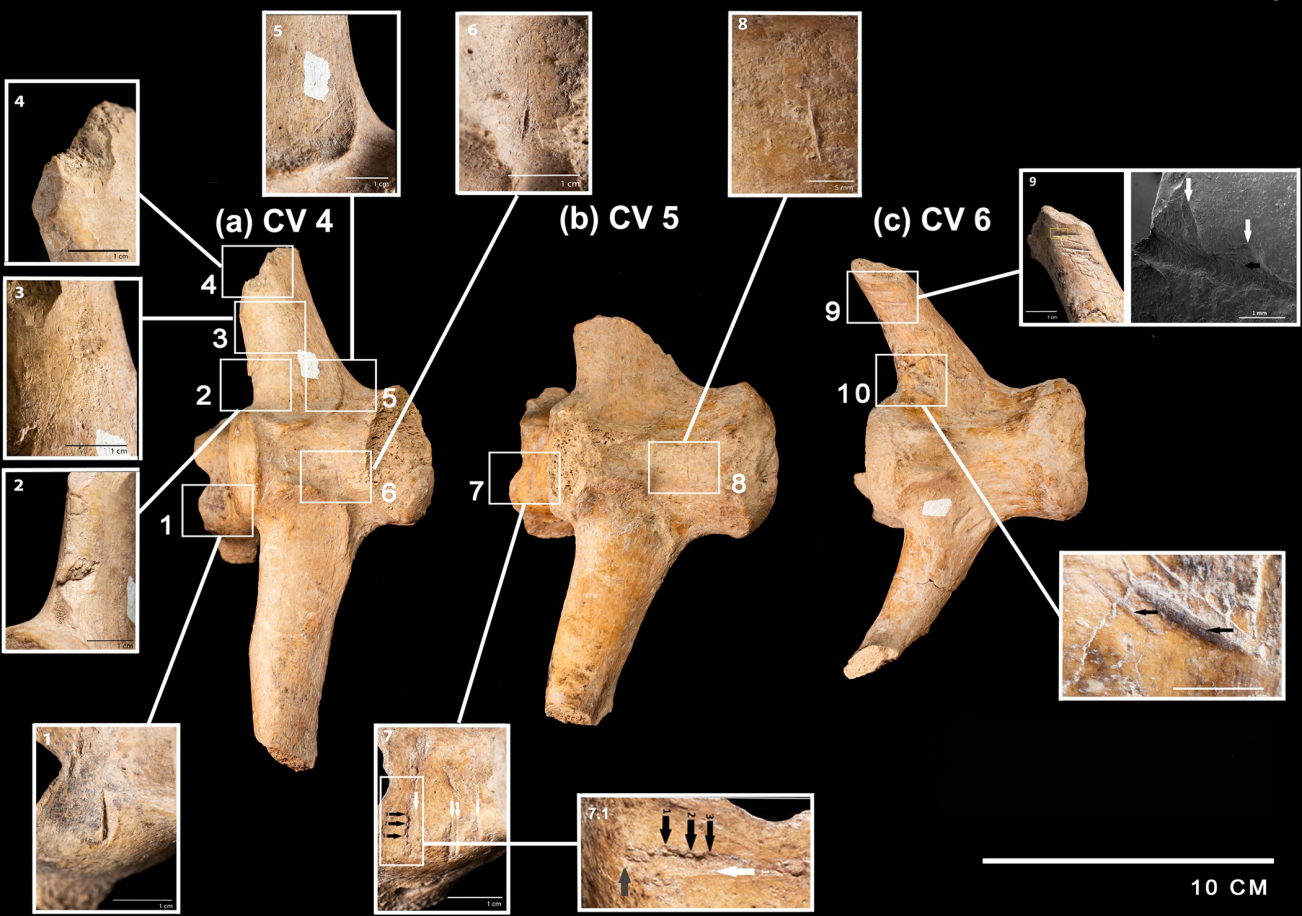
Distribution of marks found in caudal vertebrae of the CRS-10 specimen: a CV4 (1 short, deep, and V-shaped wide linear mark, 2 and 4 chop marks, 3 scraping mark [black arrow], 5 and 6 elongated V-shaped cut marks); b CV5 (7 cut marks in neural process [white arrows] and hertzian cones [black arrows], 7.1 detail of a cut mark with an asymmetrical cross-section and raised shoulder due to bone accumulation hertzian cones [white arrows]; micro-striations [black arrows] and supine line [grey arrow], 8 linear mark in vertebra body); c CV6 (lineal marks identified on the distal portion of the left transverse process of CV6. 9 detail of the main mark obtained by scanning electron microscopy exhibiting hertzian cones [white arrows] and microstriations; 10 cut marks with internal microstriations [black arrows].

**Fig 8:**
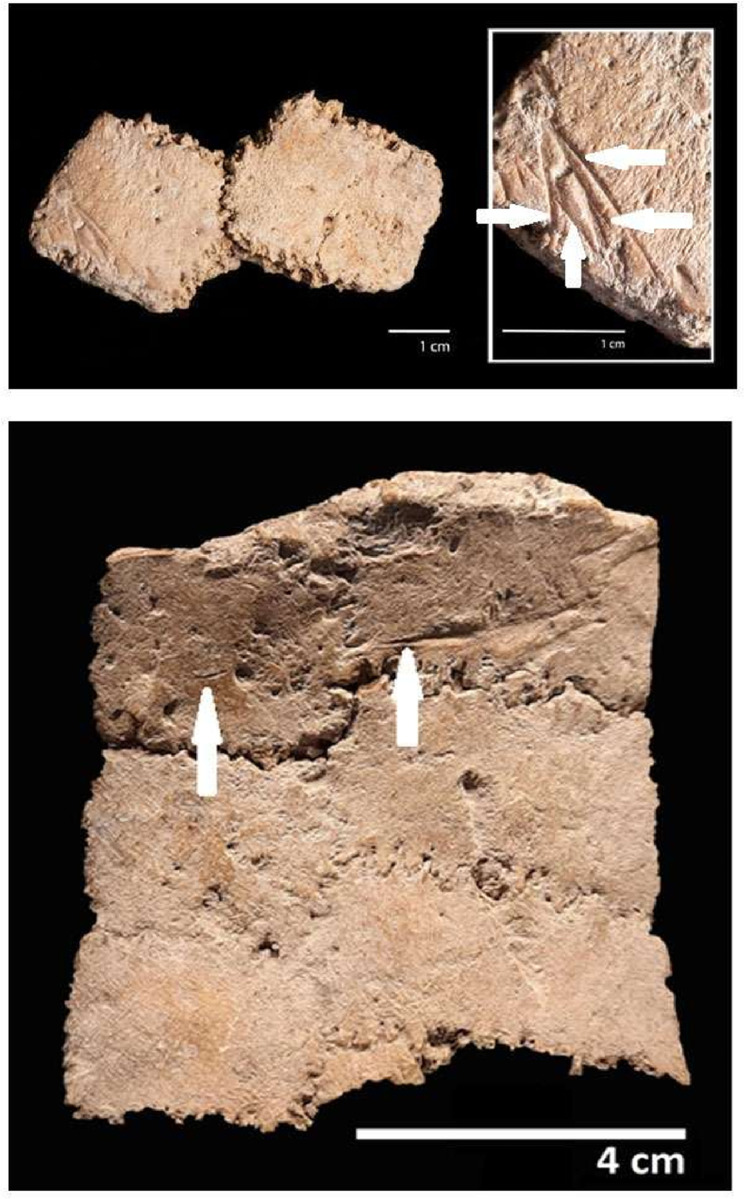
Lineal marks (white arrows) identified on the inner portion of the caudal rings.

A total of thirty-two cut marks were identified in the posterior caudal portion of the specimen. They are found in the pelvis (Fig 6), caudal vertebrae (Fig 7) and in the osteoderms of the caudal rings (Fig 8). In the pelvis, there are marks on the acetabular edge of the inner face: two main linear V-shaped marks converge towards the acetabular edge while in the upper one a shoulder effect is observed. In the lower one, hertzian cones are present (Fig 6). In three caudal vertebrae, there are cut marks in the ventral position, shown in detail in Fig 7.

The caudal vertebra 4 (CV 4) (Fig 7A) has in the right portion of the neural process a short, deep V-shaped wide cut mark with displaced bone (Fig 7A 1). In the left transverse process, there are two marks whose pattern involves cortical structures, creating a cut plane parallel to the bone axis (Fig 7A 2 and 4). In the ventral portion of the left transverse process, a cut mark with a sub-parallel path to the process axis is presented (Fig 7A 3) along with a V-shaped elongated linear mark (Fig 7A 5). Finally, an elongated V-shaped cut mark is present in the ventral portion of the vertebra body (Fig 7A 6).

In the caudal vertebra 5 (CV 5) (Fig 7B), there are two sets of cut marks and an isolated linear mark. In the right portion of the neural process, a set of deep longitudinal incisions is recorded, and arranged in a concentrated and superimposed manner with the presence of hertzian cones and microstriations and supine line (Fig 7B 7). The second group is concentrated on the neural process limited in the anterior portion by a short and deep cut mark. This effect resulted in the loss of cortical bone extending to the posterior portion up to two parallel V-shaped cut marks (Fig 7B 7). A third cut mark transverse to the caudal axis of the CV 5 body is also present (Fig 7B 8).

In caudal vertebra 6 (CV 6) (Fig 7C), there are twelve cut marks arranged in two groups. The first set is presented perpendicular to the main axis of the left transverse process arranged in a parallel and subparallel way (Fig 7C 9). The second set is represented by three cut marks located at the proximal end of the left transverse process that converges towards the center of the vertebra body. One of these marks is V-shaped and has an elongated and shallow appearance. The other marks are short, deep with microstriations and V-shaped (Fig 7C 10).

**Fig 9:**
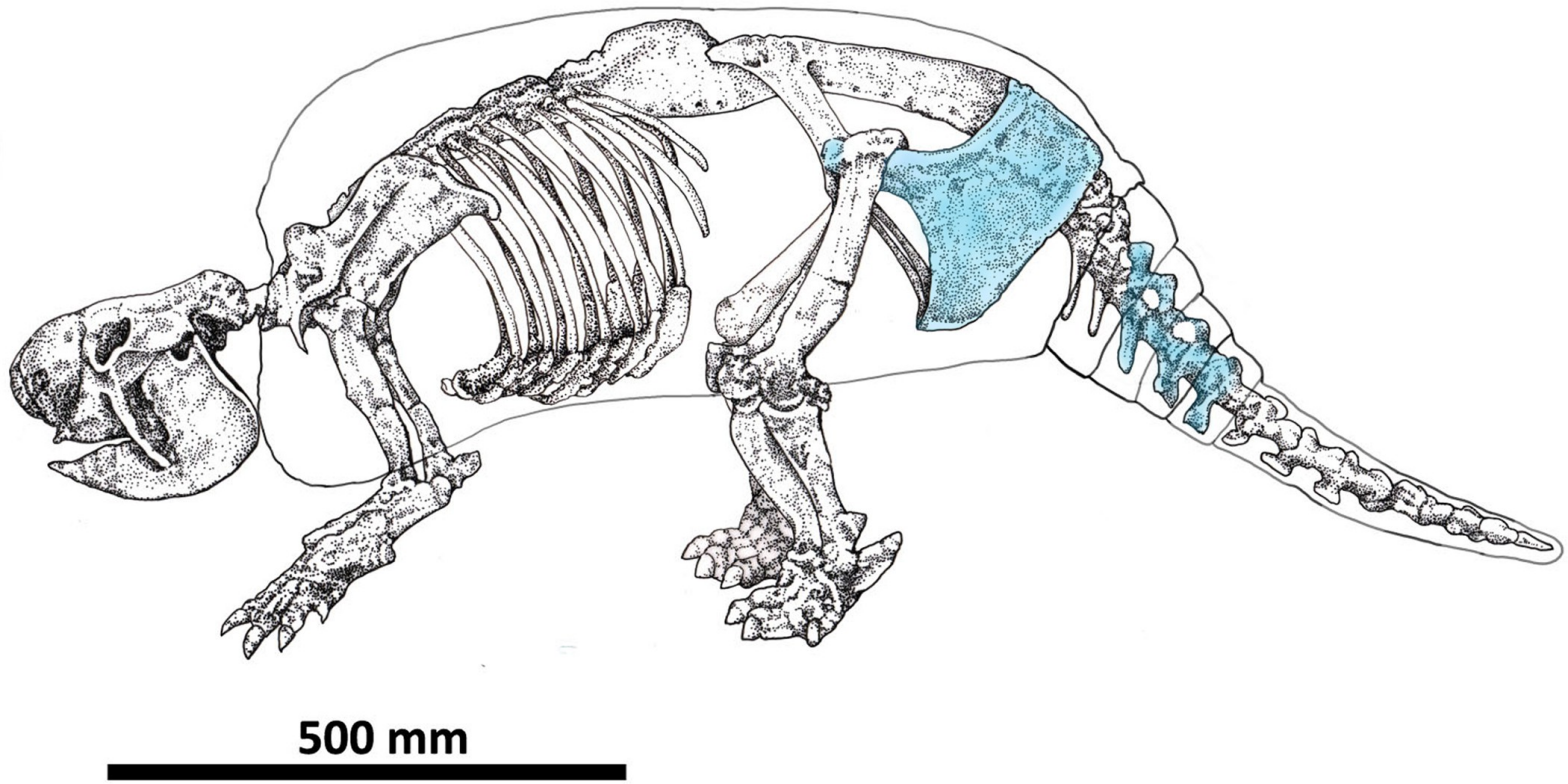
Drawing of a *Neosclerocalyptus* skeleton highlighting cut-marked skeletal elements in light blue found at the CRS-10 specimen.

**Fig 10:**
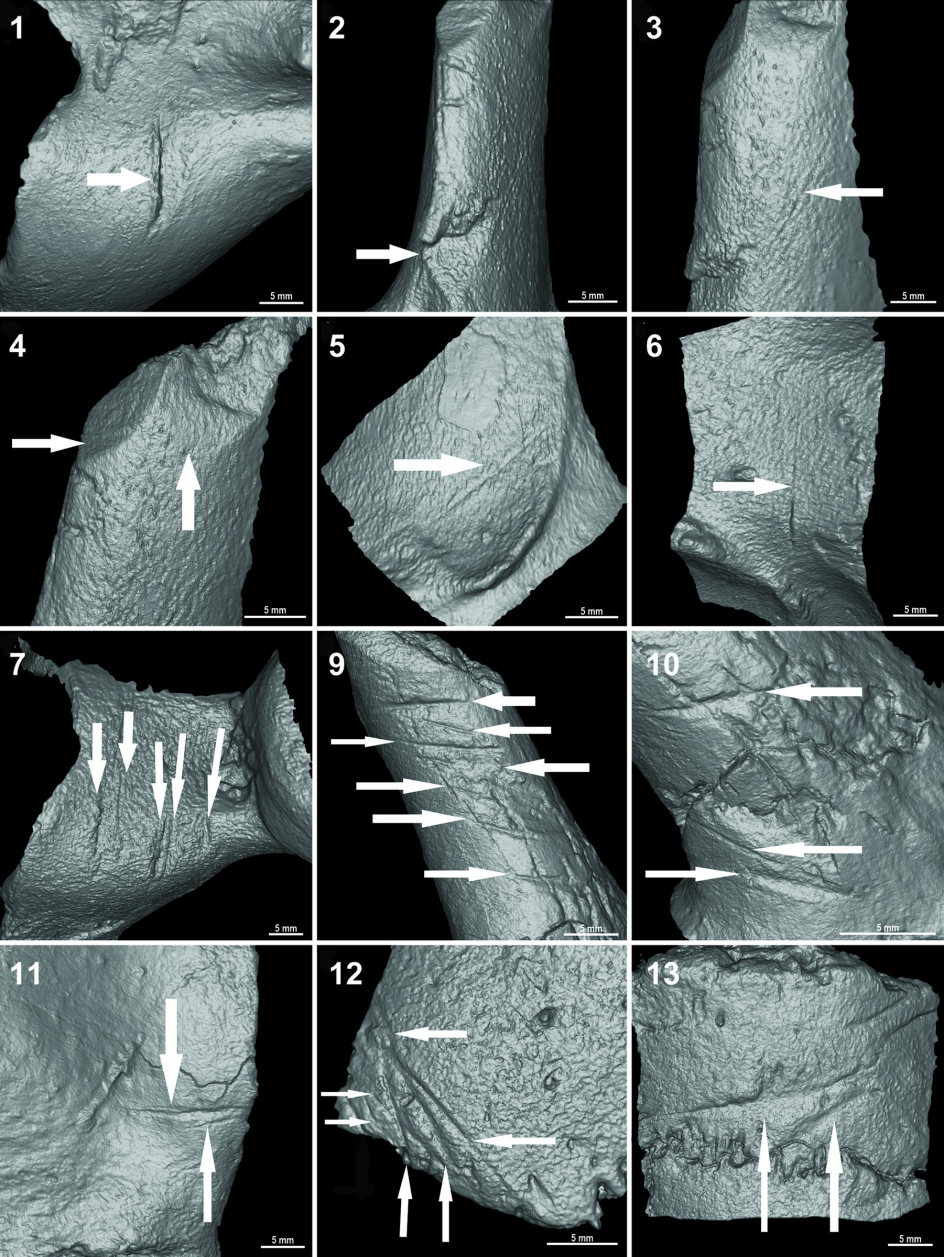
Three dimensional reconstructions of cut marks found in the *Neosclerocalyptus* sp CRS-10 specimen.

Associated with the modifications observed in both pelvis and three caudal vertebrae, cut marks were found on the inner face of the osteoderms belonging to the caudal rings. A first set is represented by the presence of seven marks which are arranged in a parallel and subparallel way, one of them V-shaped on the vertices (Fig 8A). On the other hand, a second caudal ring has two transversal marks, both V-shaped (Fig 8B). In Fig 9, the cut marked skeletal elements found in the CRS-10 specimen are represented. Finally, high-resolution 3D reconstructions of the cut marks found in different anatomical elements are presented in Fig 10. The 3D help one visualize more clearly the wide range of cut marks found in the CRS-10 specimen.

Overall, the cut marks found at the CR-10 specimen on bone surfaces suggest a pattern of distribution not at random (i.e., related to behavior) and, in several cases with signs of human-made marks (V-shaped rather than U-shaped marks, internal linear striations, among others).

### Quantitative analysis of the cut marks

**Fig 11:**
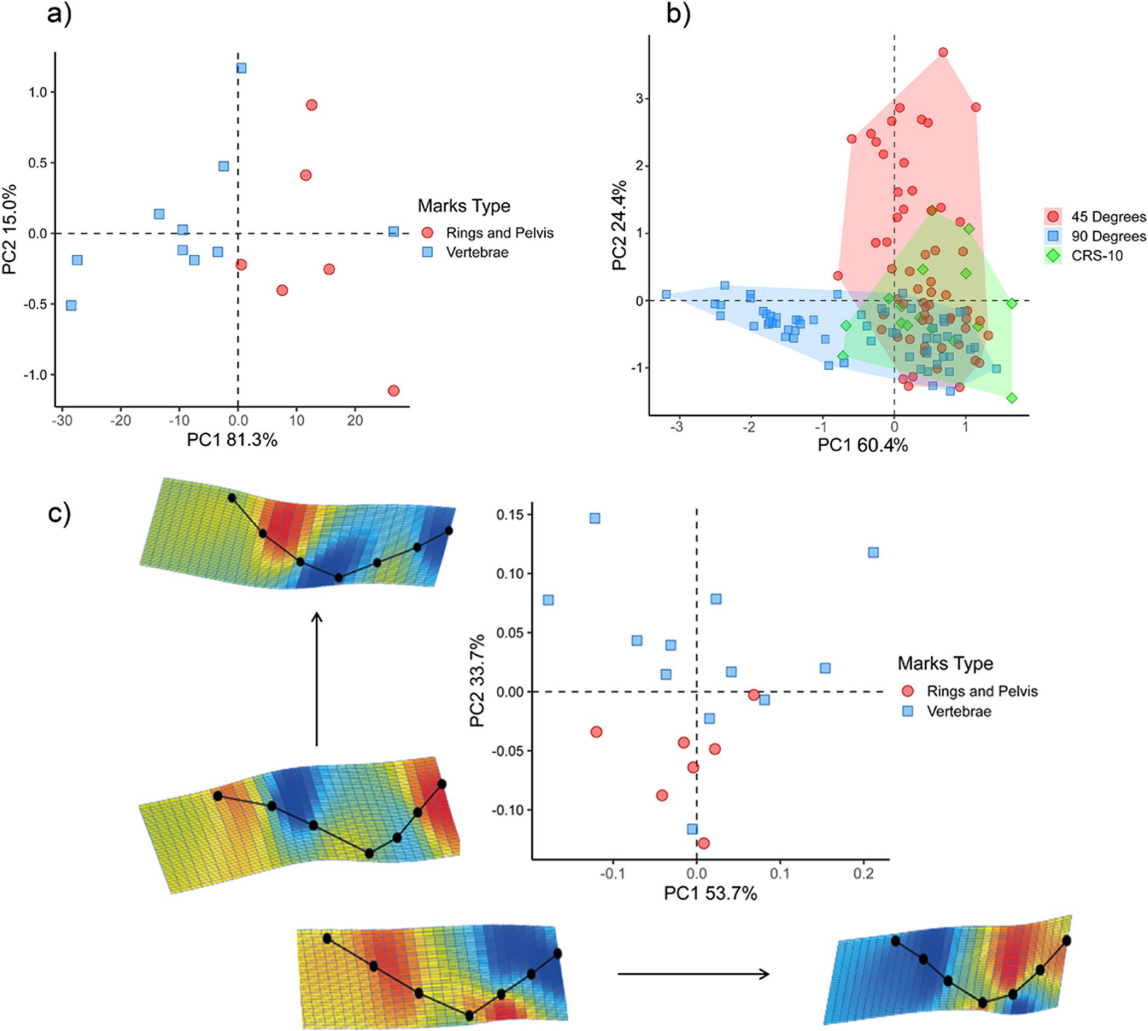
Results of the PCAs obtained from the investigation of morphometric and 2D geometric morphometric data derived from cut mark cross-sections from the CRS-10 specimen. Scatterplot of the first two PCs (93.6% of the total variance) for the morphometric analysis of seven linear measurements obtained from 16 cut mark cross-sections from the CRS-10 specimen discriminated by anatomical part (rings/pelvis and vertebrae) (a). Scatterplot of the first two PCs (84.8% of the total variance) for the morphometric analysis of seven linear measurements obtained from 16 cut mark cross-sections from the CRS-10 specimen and experimentally by Courtenay and colleagues [60] at two different cutting angles 45° (N = 60) and 90° (N = 60) (b). Scatterplot of the first two PCs (87.4% of the total variance) for the cut mark cross-section shape variation by type (N = 19) (rings/pelvis and vertebrae) (c). Shape changes are represented as TPS-grids showing deformations for the PC positive/negative scores and color-coded Jacobian expansion/contraction factors which measure the degree of local expansion or contraction of the grid. Yellow to red factors indicate expansions and light to dark-blue factors indicate contractions. The green color indicates areas with few or no changes. Scale factor of the grid 0.1 units.

Following Courtenay and colleagues [60], we obtained 7 linear measurements from selected marks (N = 19) found at the CRS-10 specimen (see Materials & Methods) to perform quantitative comparisons (Table 2, S3 Table). After computing a PCA, we removed from the linear morphometric analysis three cut marks located on the pelvis (PL2_2) and the CV 4 (CV4_1 and CV4_ 2), given their large size that caused statistical outliers in the scatterplots (see S4 Fig). The first two PCs explain 93.6% of the total variance (Fig 11A). The PC1 suggests a pattern that differentiates marks on the rings and pelvis (positive scores) from marks on the vertebrae (negative scores). The PC loadings (Table 3) suggest that all variables, with the exception of RDC presented high correlation coefficients. The PC2 presents an unclear pattern that differentiates marks on the basis of high values (positive scores) versus low values (negative scores) of the right depth on the incision convergent (RDC).

**Table 2:** Descriptive statistics of seven linear measurements obtained from 2D pictures of cut marks cross-sections from the CRS-10 specimen compared to two datasets obtained at two different cutting angles 45° and 90°from Courtenay and colleagues [60].

	WIS	WIM	WIB	D	LDC	RDC	OA
	This study						
Median	1.068	0.740	0.406	0.283	0.673	0.5513	122.368
Minimum	0.420	0.270	0.140	0.070	0.220	0.2600	94.000
Maximum	3.070	2.120	1.220	0.830	2.670	1.0000	149.000
Std. Dev.	0.629	0.445	0.264	0.172	0.568	0.1990	16.436
	Experimental dataset 45°						
Median	1.221	0.701	0.223	0.223	0.305	1.048	119.950
Minimum	0.310	0.160	0.060	0.070	0.100	0.260	92.360
Maximum	2.890	1.640	0.500	0.490	0.700	2.530	141.310
Std. Dev.	0.620	0.342	0.101	0.114	0.152	0.552	10.739
	Experimental dataset 90°						
Median	0.617	0.370	0.148	0.264	0.393	0.438	98.279
Minimum	0.280	0.180	0.070	0.090	0.170	0.200	36.440
Maximum	1.080	0.660	0.330	0.530	0.620	0.660	143.970
Std. Dev.	0.200	0.120	0.057	0.098	0.103	0.107	27.878

**Table 3:** PC loadings of seven measurements from 16cut marks of the CRS-10 specimen.

Measurement	PC1	PC2
WIS	**-0.988**	-0.135
WIM	**-0.990**	-0.115
WIB	**-0.986**	-0.077
D	**-0.966**	0.227
OA	**-0.973**	0.022
LDC	**-0.902**	-0.189
RDC	0.262	**-0.962**

In bold high correlation coefficients.

The PCA comparing the marks of the CRS-10 specimen with those obtained experimentally by Courtenay and colleagues [60] at two different cutting angles 45° (N = 60) and 90° (N = 60) (Fig 11B and Table 3) shows that PC1 (60.4% of the total variance) differentiates the cut marks obtained at 45°and 90° illustrating the findings of the original study [60]. Importantly, the marks from the CRS-10 specimen are evenly distributed across the multivariate space within the range of those obtained at 45°and 90° respectively. The factor correlations (Table 4) indicate that such a pattern is influenced by measurements with high and positive loadings (i.e., WIS, WIN, WIB, and LDC), that is, changes in the width of the marks. The PC2 (24.4% of the total variance) tends to differentiate marks at 45° compared to the marks at 90° and those from the CRS-10 specimen. This distinction is related to the depth of the incision (D) and the opening angle of the incision (OA), which presents high positive and negative loadings, respectively. The most conspicuous finding is that the diversity of the cut marks found at the CRS-10 falls within the range of variation of the cut marks made by humans.

**Table 4:** PC loadings of seven measurements from 16 cut marks of the CRS-10 specimen and those derived from the experimental datasets published by Courtenay and colleagues [60].

Measurement	PC1	PC2
WIS	**0.954**	0.175
WIM	**0.978**	0.168
WIB	**0.851**	0.129
D	0.582	**-0.786**
OA	0.692	-0.432
LDC	**0.874**	0.105
RDC	0.247	**0.906**

In bold high correlation coefficients.

Additionally, the results of the 2D GMM analysis (S4 Table) for the first two PCs (87.4% of the total variance) show a distinction between close and open V-shaped marks along PC1 where the first are located at the right of the plot (positive scores) and the latter at the left (negative scores). The shape changes represented as TPS deformation grids and color-coded Jacobian expansion/contraction factors show differences in cut mark depth and opening angle, that is, contraction of the left depth of the incision convergent and expansion of the right depth of the incision convergent as well as changes in the depth of the incision (Fig 11C). The PC2 detected the same pattern observed through the investigation of linear measurements regarding the differentiation between cut marks located at the vertebrae and on the rings and pelvis. Whereas the marks located on the vertebrae present close angles and lesser depths of the incision, the marks located at the rings and pelvis present the contrary pattern as well as differences in the degree of the Jacobian expansion and contraction. Jointly, the quantitative analyses of CRS-10 specimen cut marks revealed a clear differentiation by anatomical part, angle, and depth of the incision and shape of the marks, and importantly, strong similarities to those marks obtained through the experimental approach.

The results of the MANOVA and LDA analyses are presented in Tables 5 and 6 and S5 Fig. Whether including or not the OA variable, the MANOVA results indicates that both kinds of cutting marks (cuts and slices) have significantly different means. Likewise, the LDA results show that the classification/misclassification matrix was able to correctly assign between 91% (95% CI 0.8509–0.9536) and 93% (95% CI 0.8634–0.9501) of the samples to their correct group. Interestingly, the exclusion of OA improves both the mean differences and the percentages of correct classification. The histogram with the LDA scores for leave-one-out cross-validation (S5 Fig) shows that the reclassification of the CRS-10 specimen cut marks into two categories (slicing and cutting) does not alter the differentiation pattern between the experimental cut mark types. This is reinforced by the high proportion of trace explained by the LD1 (0.100). These results are very similar to those reported by Courtenay and colleagues [60] indicating that the CRS-10 cutting marks are morphometrically similar to those human-made.

**Table 5:** MANOVA results for the comparison between CRS-10 and experimental slicing (45°) and cutting (90°) marks.

Measurement	F	P
All measurements	31.4	<0.0001
Measurements excluding OA	35.1	<0.0001

**Table 6:** LDA confusion matrix results and average % of correctly classified cut marks combining the CRS-10 and experimental cutting marks.

Measurement		45°	90°	% Correct	95% CI	P
All measurements	45°	60	5			
	90°	7	64	91.18	0.8509–0.9536	<0.0001
Measurements excluding OA	45°	59	4			
	90°	5	65	93.42	0.8634–0.9501	<0.0001

## Discussion and conclusions

### Paleoenvironmental scenario, deposit context and taphonomic inferences

The observed characteristics of the sediments where the CRS-10 specimen was found suggest a fluvial paleoenvironment within a point/bar as previously proposed [71]. Overall, the sedimentological analyses suggest that the possible deposition context of the CRS-10 specimen occurred in environments predominated by semi-arid climates with alternating dry and wet seasons, as suggested by the laminate filler inside the carapace and the carbonate nodules in the sedimentary stratum. The laminates found inside the carapace reveal that at the beginning of the burial, the first sediments were deposited by suspension, revealing that these structures were in the presence of water (wet season). It could come from sporadic rains where the concave part of the carapace acted as a vessel where water and suspended sediment accumulated. After three events of this type (as evidenced by the laminates), there was a massive burial given by eolian sediments of a massive structure. It is possible that these deposits were not formed by dunes given that the Eloisa member does not have the typical interlocking structures and the bone surfaces have no evidence of abrasion by sand impact.

Another evidence of the prevailing climatic conditions that occurred during the burial lies in the development of the mottled oxide of Fe and Mn on the bones. These oxides typically only precipitate under alkaline conditions and water scarcity [86,87]. These conditions would coincide with the development of the observed sedimentary structure, that is, lateral accretions, where the fluvial course moves away from the location of the bones thus lowering the availability of moisture.

All of these inferences coincide with previous paleoenvironmental and paleoclimatic reconstructions regarding the prevailing conditions in the Pampean region during the LGM, that is, the predominance of semiarid climates and reduced temperature, moisture, and precipitation [88,89]. Additionally, palaeontological analysis on the morphological adaptations occurred in *Neosclerocalyptus* spp. genera in the Southern Cone suggest that the nasofrontal structures are adapted to semiarid climates indicating a correspondence with our sedimentological and taphonomic inferences as well as with previous paleoenvironmental reconstructions in the study region [90].

Several indicators, such as the little or non-disarticulation of some skeletal parts, the lack of movement of some elements regarding their anatomical position (e.g., pelvis and vertebrae) (Fig 3), and the very good preservation of the cortical surfaces suggest little postdepositional distortion of the fossil assemblage recovered, an undisturbed and reliable deposit context, and a fast burial scenario. This is further supported by the degree of weathering present in the CRS-10 bone elements, which presented 0–1 stages in Behrensmeyer´s scale [91]. The curvature that describes the joint *in situ* of the caudal vertebrae (Fig 3) added to the position inside the carapace allows us to suggest a certain degree of contraction due to dehydration of the tendons and muscles. This caused a subtle postmortem movement of the vertebral and caudal tube located inside the carapace discarding an opisthotonic "agonizing" posture [73].

The lack of evidence of pits and furrows indicating carnivore/rodent activities allow us to infer the minimal action of distinct taphonomic agents like animal scavengers. Specifically, rodent activities are difficult to be related to the marks found since they are w-shaped, have a flat bottom, and are found in pairs. We found no evidence of these indicators in the fossil bone surfaces. Likewise, carnivore activities are not a likely source of the cutmarks found because tooth marks left by carnivores are usually U-shaped and appear in the form of pits followed by grooves producing marks decreasing in intensity as they move away from the point of origin. We neither found serrated edges on bone surfaces produced by the chewing of the epiphysis. In addition, we don’t detect bone wear related to bone sucking typical of some canids and felids. Apart, the anatomical disposition observed during the specimen recovery suggests that there was little or no disarticulation, especially along the caudal vertebrae. This condition would not be consistent with the action of additional agents such as trampling or bearing, since they tend to produce the displacement of the anatomical units from their natural articulation, a product of the external mechanical force exerted, and shallow marks placed randomly on bone surfaces.

### Linear marks and human agency

The position and distribution of cut marks on bone surfaces can’t be satisfactorily explained by distinct factors such as trampling, bearing, the distorting effect of diagenesis, and the action of carnivores, rodents, among others. The paleoenvironmental reconstruction and the deposit context revealed that the CRS-10 specimen remained mostly *in situ* after the burial event with little postdepositional disturbance. In this sense, the morphology observed in some of the cut marks described in this work presents a series of characteristics attributable to cut marks originated by anthropic action. For example, the cut marks with some attributes like hertzian cones, shoulder effect, displaced bone, supine line, etc., suggests that people cut the bone when it was fresh. Some marks presented an asymmetrical cross-section, with a steeper slope, where the development of hertzian cones can be observed [92] along with bone accumulation [93]. The hertzian cones [92] are small lateral elevations to the main groove of triangular morphology, produced by the differential pressure exerted on the bone surface when cutting and by the resistance of the bone surface to cutting (Fig 7B and 7C) [93]. The presence of hertzian cones allows establishing the directionality of the cut while the presence of supination at the end of the cutting marks allows identifying the end of the path (Fig 7B) [92]. On the other hand, the displaced bone may accumulate on an elevated shoulder of bone that runs along the linear mark, which, in the absence of abrasion, may remain *in situ* (Fig 7B). Other attributes are represented by the presence of micro-striations towards the interior of the analyzed cut marks (Figs 7A 1–6, 4B 7 and 4C 10), which can be attributed to mechanical action of trampling, bearing or by the action of retouched edges of lithic instruments [68]. However, the micro-striations derived from trampling and bearing differ from the cut marks since are less deep, shorter, and less frequent without a clear association with insertion areas of tendons or muscles [62,69]. Lastly, pseudolinear marks parallel to the primary stretch marks, named as shoulder effect [69] (Fig 6), are also observed. This effect is produced by irregularities in the edge of the instrument used at the time of cutting [68].

In addition, the location of the cut marks in different anatomical units, such as pelvis, caudal ring osteoderms, and caudal vertebrae shows the configuration of a butchering sequence that can be understood from an anatomical perspective, where large muscle packs are anchored from the pelvic waist (synsacrum) to the transverse processes of the caudal vertebrae. This musculature represents nearly 70% of the total volume of muscle mass of these animals [93], so its use implies the acquisition and consumption of a significant amount of meat. In this regard, the presence of these cut marks indicating muscle use can represent different stages of a butchering sequence of the muscle packs of the pelvic waist and tail [94]. Given the size of the specimen, transportation would not have been a probable option [94]. In addition, in the caudal vertebrae a non-random pattern of cut marks distribution is described, since the cut marks are specifically concentrated on the ventral face of the left transverse processes of CV 4 and CV 6, in the left portion of the neural process in CV 4 and CV 6 and in the right anterior portion of the body in VC 4 and VC 5 (Fig 8) probably indicating preferences in the use/consumption of specific areas along the caudal vertebrae especially taking into account both the position of the specimen (i.e., ventral) and the correspondence with marks located in other parts (i.e., rings). Importantly, the pattern evidenced regarding the size/shape differentiation of marks located at distinct anatomical parts–rings/pelvis versus vertebrae–revealed by the morphometric analyses also deserves mention. This morphometric distinction is likely related to the differential pressure exerted by lithic tools on bone surfaces with distinct bone densities and cortical bone thickness. Precisely the shape-changes revealed differences in the angle and depth of the incision in both anatomical elements supporting this interpretation. Such a differentiation could be related to diverse butchering and processing strategies and makes sense in terms of the distribution of meat in such skeletal parts. These inferences reinforce the hypothesis of the existence of butchering sequences reflecting behavioral patterns that are difficult to be explained beyond human agency.

The quantitative analyses representing an additional and independent line of evidence strongly support the anthropic nature of the marks found on the CRS-10 *Neosclerocalyptus* specimen. The comparison between the cut marks found in the fossil specimen and cut marks obtained experimentally at two different cutting angles, 45° and 90°, suggest remarkable similarities both in terms of the distribution across the PCA morphospace and the shape changes. Interestingly, the cut mark cross-section 2D shape changes evidenced in the present study are almost identical to those observed in the referenced study [60]. Both kinds of cut marks share several morphometric attributes revealing a common factor regarding the size, shape, and distribution of cut-marks made on animal bones. Likewise, the MANOVA and LDA results indicate that the CRS-10 cut marks can be morphometrically divided into two main groups: cuts and slices, similar to the experimental dataset suggesting that the late Pleistocene humans processing the fossil bones used butchering strategies that resemble the ones used in the experiment by Courtenay and colleagues [60].

Overall, our results support recent studies which used state-of-the-art quantitative methods to investigate hominin behavior through cut marks and notably to differentiate human from non-human agents [60,72,95–97]. In the present case, the multiple and independent lines of evidence investigated solidly support the human origin of the cut marks found in the CRS-10 specimen.

Finally, it worth mentioning that the shape of some of the marks investigated could reflect partially the intrinsic properties of bone (e.g., cortical bone thickness, bone density, etc.). However, this fact does not explain the similarities between the cut marks found in the fossil specimen and those derived from the experimental study, and remarkably, does not account for the behavioral patterns inferred.

### Early peopling and human-megafauna interaction in southern South America

The results obtained in this work provide new empirical data suggesting interactions during the Last Glacial Maximum between humans and megafauna in southern South America, an area where there is a growing amount of reliable evidence about the exploitation and consumption of megafauna. The presence of a specimen of *Neosclerocalyptus* sp. with evidence of anthropic modifications in the northeast of the Pampean region further expands the spatial range of these interactions. Until now, reports of such interactions using distinct kinds of evidence (cut marks, burned bone, breakage patterns, etc.) have been limited to the sector located between the mountain systems of Tandilia and Ventania along with the Atlantic coast with the latter showing the earliest evidence [20–31]. The anthropic modifications described here as cutting marks are of particular interest since they allow to defend a direct interaction between humans and megafauna known as behavioral associations *sensu* [11], which indeed, taking into account previous evidence recovered in the Pampas with reliable associations, suggest a common pattern of early hunter-gatherer activities related to subsistence strategies based on megamammal exploitation [21, 50 and references therein].

The radiocarbon date obtained directly from the CRS-10 specimen 21,090–20,811 cal BP, the anthropic nature of the cutting marks described above along with its associated butchering patterns, push back the chronological frame of both human presence and human-megafauna interactions nearly 6000 years earlier than recorded for other sites in southern South America which have chronologies ranging from ~ 8000 to 15000 cal YBP [21–50]. Previous reports of archaeological sites with early dates in this region have generated controversy, and the timelines have been the subject of multiple criticisms [98–100]. Notwithstanding this, in other regions of the subcontinent (eastern South America) reliable archaeological evidence recently recovered [19], which along with the evidence presented here, allows us to envisage an emerging scenario of an older presence of humans in South America. The chronology of the CRS-10 specimen seems to be robust, as indicated by two independent proxies, the radiocarbon dates and the sedimentary information. For the latter, the specimen of *Neosclerocalyptus* sp. was found in deposits of the Eloisa Member between the deposits corresponding to the Jáuregui Member (lower deposits) dated at 31,000 years BP, and the deposits corresponding to the El Rincón Member, whose dating was determined at 17,000 years BP (upper deposits) [71]. Therefore, our chronological context is consistent with the local geology and established stratigraphy, indicating reliable radiocarbon dates from both the specimen and the lower floor of the deposits. Additional ^14^C assays, currently in course, will provide further details regarding the radiocarbon chronology of the specimen here investigated.

The timing of the initial peopling of the Americas is a matter of controversy and debate, and several findings and lines of evidence suggest earlier dates for the first human entry than the commonly proposed. Our results, fit with recent findings showing evidence of human occupation between 20,000 and 30,000 years ago in Central and South America [19,101–104] as well as North America [105,106]. Finally, a recent study [107] has suggested that the upper limits of the chronological range for megamammal species commonly exploited by humans in South America date back to 18 k cal BP while the archaeological signal goes back to16 k cal BP, indicating, along with the evidence provided by others sites mentioned above [19], that the archaeological signal of the human occupation of southern South America is consistently and progressively reaching the Last Glacial Maximum. Taken together, these evidences allow envisages a new perspective regarding the timing of the initial entry of humans to the Americas. Interestingly, such a study also shows that the Pampean region presents both a high frequency of late Pleistocene archaeological sites and high values of megafaunal species richness [107]. This fact indicates that our site has a high potential to preserve evidence of human-megamammal interactions.

Our findings are inconsistent with the chronological frame established for the earliest human occupation of southern South America, which had been proposed to date back to ~16,000 cal BP [27,107–110]. Remarkably, a new study [111] has shown reliable evidence of human occupations in Patagonia at 17.3 k cal BP indicating earlier dates for the initial peopling of southern South America. Despite traditional peopling models tending to support a later entry of humans to southern South America, it is not ruled out the possible presence of humans and their associated cultural evidence well before 16 Kya. In this context, our results support the growing archaeological evidence indicating an earlier date for the initial peopling of the Americas and the southern Cone in particular [19,101–103].

## Limitations and future prospects

The fossil and archaeological record provides a fragmented and incomplete portrait of the processes operating in the past. Accordingly, the evidence used in our interpretations is not free of a range of biases that are difficult to be controlled even if we count on solid evidence, robust chronological contexts, and fairly complete archaeological and fossil records. Given the nature of the evidence presented (i.e., behavioral associations), it is necessary to perform a critical assessment of the results obtained so far and acknowledge some of the remaining work that would be needed to complement our findings. First, we need to establish a stronger link between fossil bones with cut marks and the archaeological record. Given that the archaeological excavation only involved a small part of the site (2 x 2 meters), it is possible that during subsequent excavations of the complete site, we can recover much more materials, including those related directly to the archaeological record. Importantly, as we mentioned above our site has a high potential to preserve evidence of human-megafauna interactions. Since an accurate and precise radiocarbon chronology is necessary to establish a reliable time frame for the deposits, starting a ^14^C dating program exploring recent methods and techniques, given the poor preservation of bone collagen, is mandatory. Fortunately, enough fossil bones are readily available to move forward with such a program. Finally, we need to perform new analyses on the cut marks using state-of-the-art imaging methods such as micro-CT scanning (μ-CT) and microphotogrametry as well as additional quantitative methods (e.g., GIS-based methods). Future research, including a large archaeological excavation, denser radiocarbon dating, and additional analyses on both bone surfaces and cutting marks will certainly add further support to our findings and help in ruling out some of the factors that we have identified as potentially confounding or not sufficiently backed up by the available evidence.

## Supporting information

S1 Fig Following Courtenay et al [60] linear measurements (taken from each cut mark cross-section—WIS, WIN, WIB, D, OA, LDC, RDC—and 2D landmarks LM) were used in the morphometric and geometric morphometric analyses (LM1-LM7). (TIF)

S2 Fig Calibration curves (curve SH cal20) for the two ^14^C dates obtained in the present study for both the fossil specimen (A) and bivalve mollusks found at the Jáuregui member (B).
(TIF)

S3 Fig Chemical composition obtained from the use of X-ray energy dispersion spectrometry. (TIF)

S4 Fig Scatterplot of the first two PCs (84.8% of the total variance) for the morphometric analysis of seven linear measurements obtained from 19 cut mark cross-sections from the CRS-10 specimen and experimentally by Courtenay and colleagues [60] at two different cutting angles 45° (N = 60) and 90° (N = 60) indicating the outliers removed in subsequent linear morphometric analysis (CV4_1, CV4_2 and PL2_2). (TIF)

S5 Fig Histogram with the LDA scores for leave-one-out cross-validation for the morphometric analysis of seven linear measurements obtained from cut mark cross-sections from the CRS-10 specimen and experimentally by Courtenay and colleagues [60] at two different cutting angles 45° (slicing N = 60) and 90° (cutting N = 60). The CRS-10 cut marks were classified into the two categories mentioned (slicing and cutting) using the posterior probabilities of the LDA. The proportion of trace explained by the LD1 was 0.100.
(TIF)

S1 Table Original reports of the radiocarbon dates obtained from the CRS-10 specimen and sediments. (PDF)

S2 Table Elemental composition of dark spots found in the CRS-10 specimen. (XLSX)

S3 Table Linear measurements obtained from the CRS-10 specimen. (XLSX)

S4 Table 2D landmarks obtained from cutmarks of the CRS-10 specimen. (XLSX)
