# Supplementary figures and images for: Anthropic cut marks in extinct megafauna bones from the Pampean region (Argentina) at the last glacial maximum
Source: PLoS One. 2024 Jul 17;19(7):e0304956. doi: 10.1371/journal.pone.0304956 (PMC11253959; doi:10.1371/journal.pone.0304956)

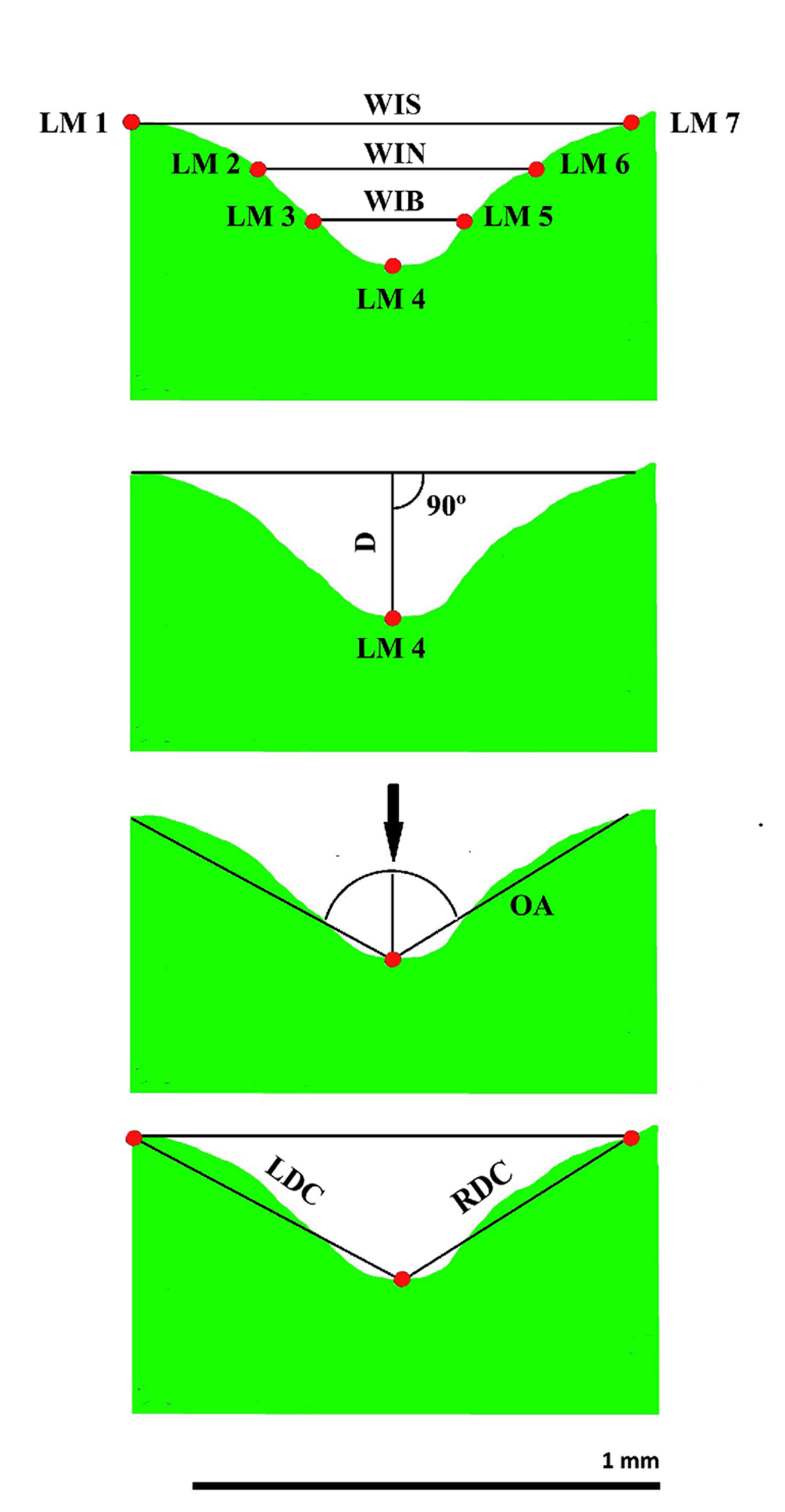

Supplement: S1 Fig — (TIF) [file pone.0304956.s001.tif]

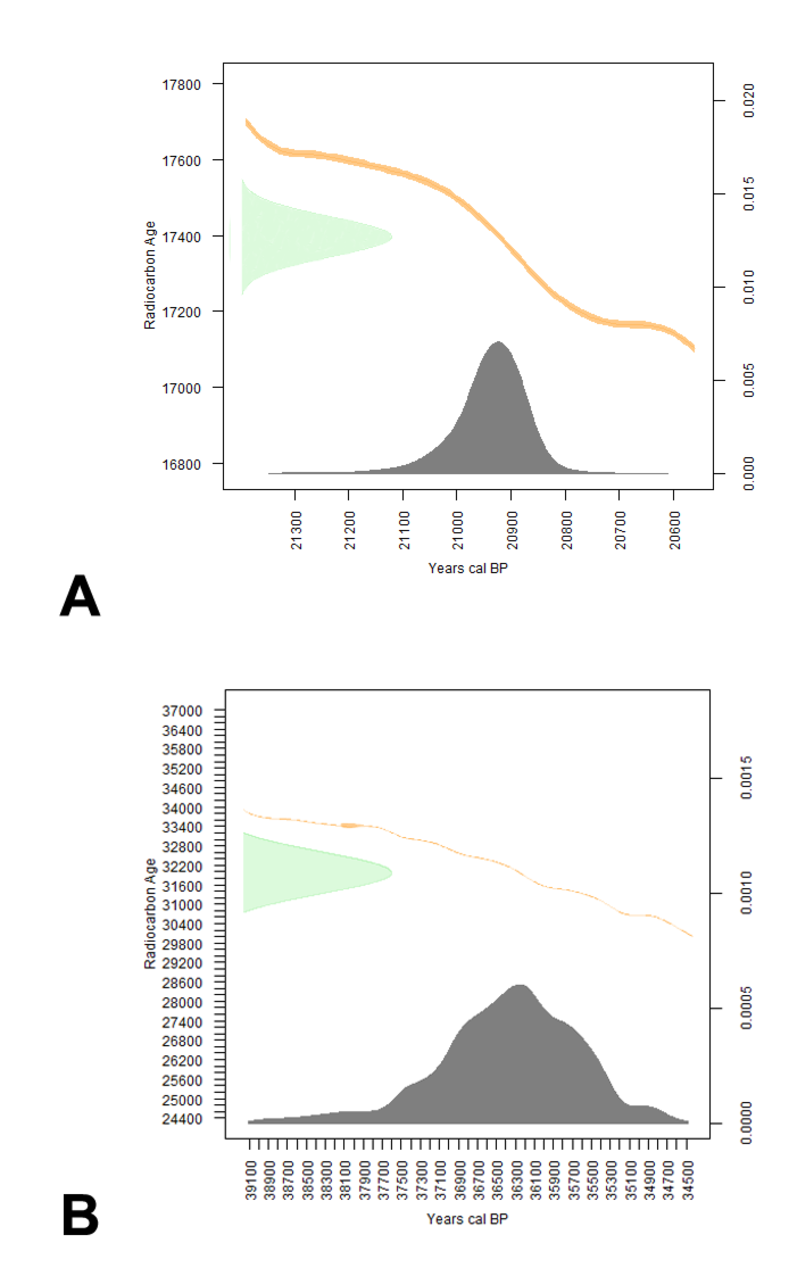

Supplement: S2 Fig — Calibration curves (curve SH cal20) for the two 14C dates obtained in the present study for both the fossil specimen (A) and bivalve mollusks found at the Jáuregui member (B). (TIF) [file pone.0304956.s002.tif]

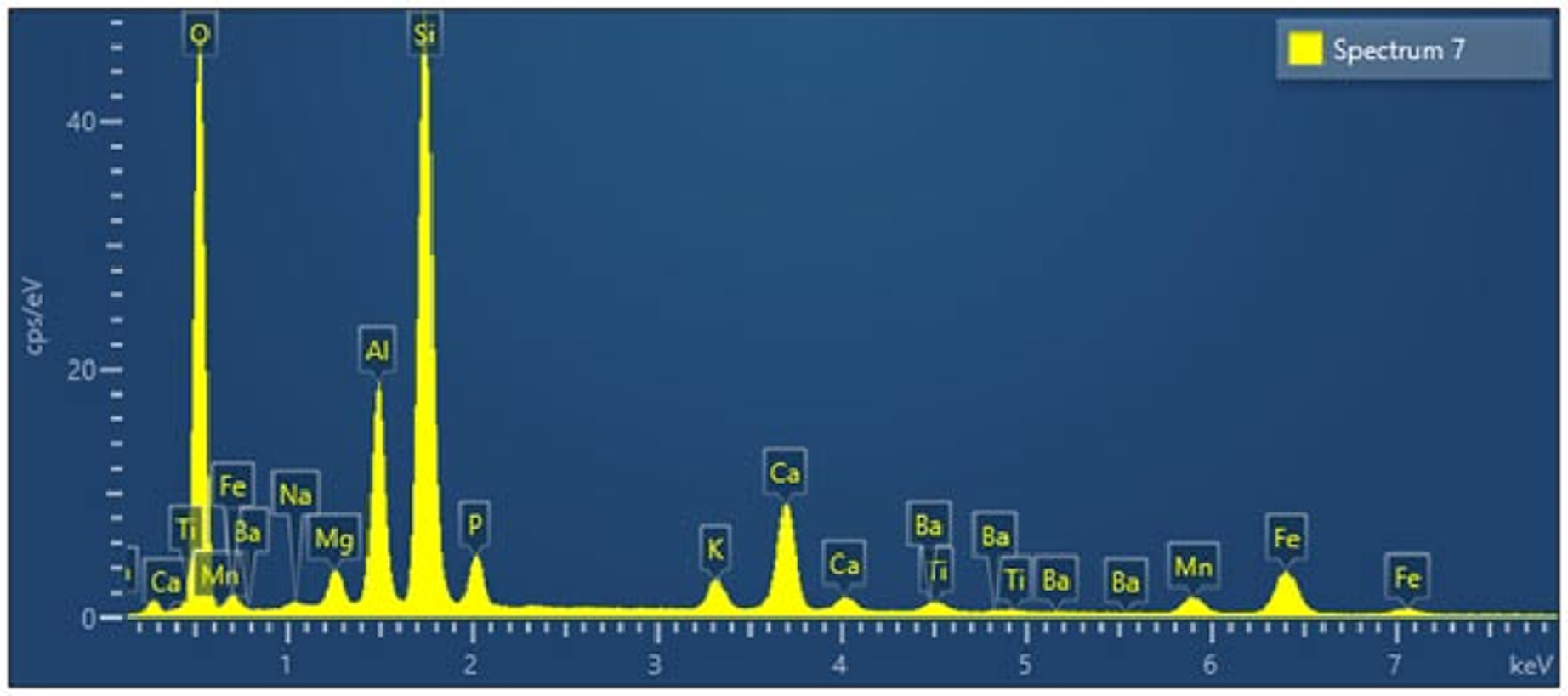

Supplement: S3 Fig — (TIF) [file pone.0304956.s003.tif]

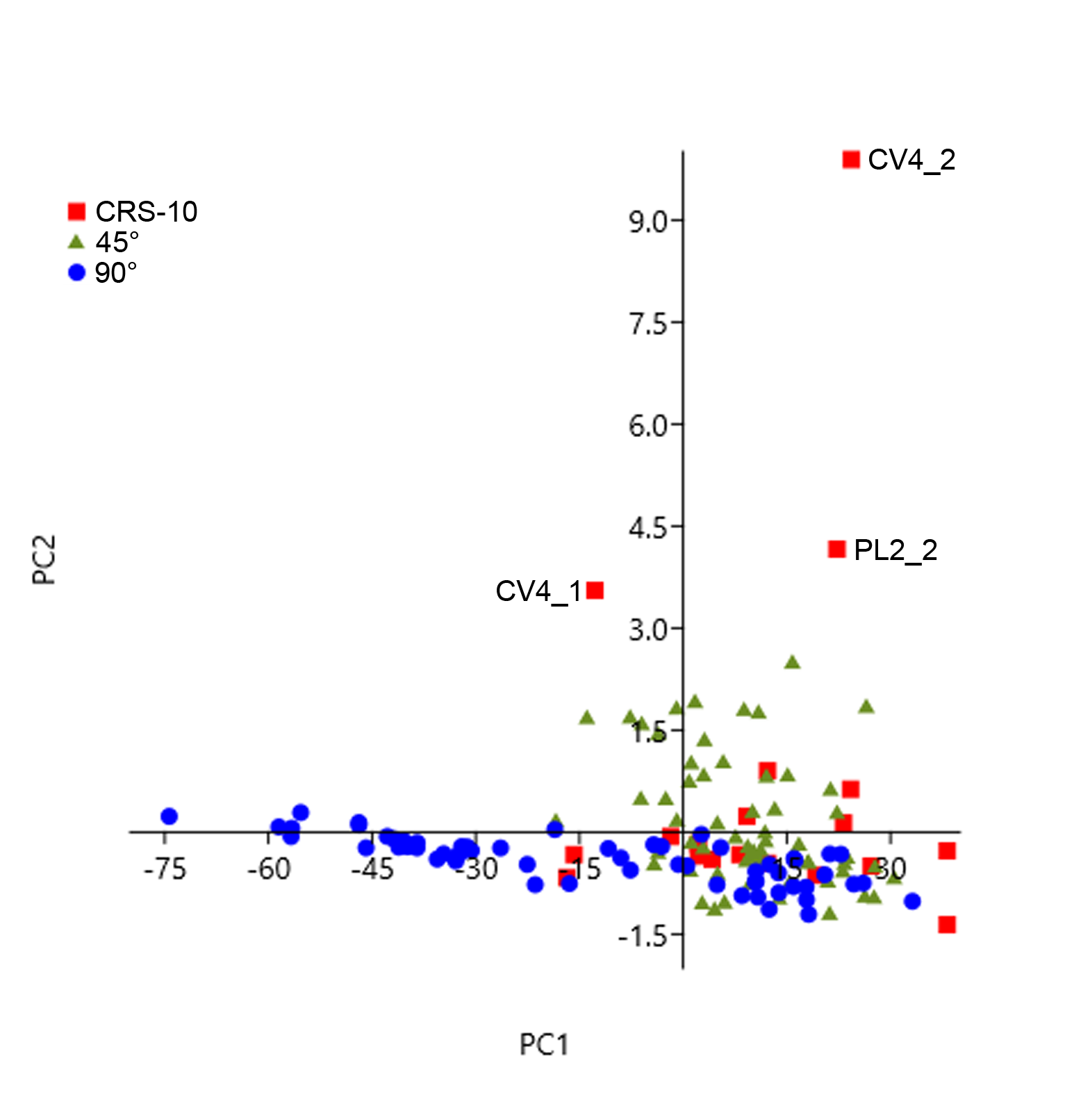

Supplement: S4 Fig — (TIF) [file pone.0304956.s004.tif]

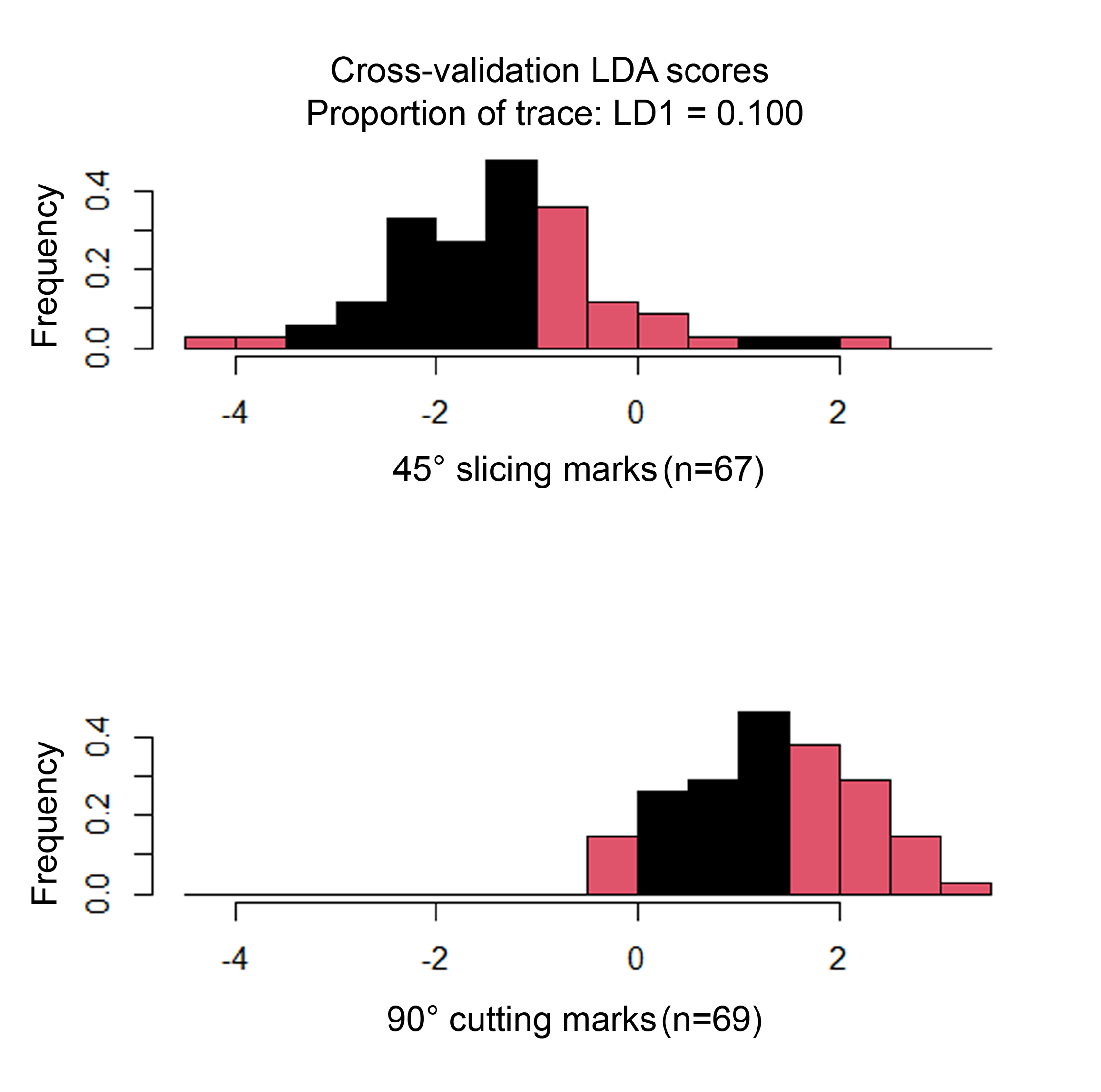

Supplement: S5 Fig — The CRS-10 cut marks were classified into the two categories mentioned (slicing and cutting) using the posterior probabilities of the LDA. The proportion of trace explained by the LD1 was 0.100. (TIF) [file pone.0304956.s005.tif]
