# Supplementary material for: Anthropic cut marks in extinct megafauna bones from the Pampean region (Argentina) at the last glacial maximum
Source: PLoS One. 2024 Jul 17;19(7):e0304956. doi: 10.1371/journal.pone.0304956 (PMC11253959; doi:10.1371/journal.pone.0304956)
Supplement: S1 Table — (PDF) [file pone.0304956.s006.pdf]

^{14}C Date 1).

Radiocarbon dating, CIRAM lab, France.

Material: bioapatite, bone fragments (vertebrae) *Glyptodontidae*

Conventional Radiocarbon Age: CIRAM-2620 17,397 ± 52 ^{14}C BP

pMC correction value: 11.47 ± 0.07

Calibration range 2σ (95.4%): 19,218 cal BC – 18,990 cal BC

OxCal v.4.4.4 Brink Ramsey (2021): c5: atmospheric data from Reimer et al (2020).

---

^{14}C Date 2).

Radiocarbon laboratory, Geological Research Centre (CIG), National Council for Scientific and Technical Research, CONICET, National University of La Plata, UNLP, Argentina.

Conventional Radiocarbon Age: LP- 3771 31,970 ± 640 years ^{14}C BP

Material: bivalve molluscs (*Diplodon lujanensis*)

Correction Factors: $\delta^{13}C$ (estimated): -8 ± 2

Error multiplier (K) = 1

Calibration for the Southern Hemisphere: SHCal20. 14C Hogg et al. 2020

Range 1σ (68.2%): 35,561 cal BP - 36,956 cal BP, relative area = 1

CALIB 8.1.0. Stuiver and Reimer, 1993.
